# Population structure of *Helicobacter pylori* and antibiotic resistance-associated variants in a high-risk area of gastric cancer

**DOI:** 10.1128/jcm.00033-25

**Published:** 2025-04-11

**Authors:** Qiu-Yu Jin, Roberto C. Torres, Chao Yang, Li- Hua He, Zong-Chao Liu, Wen-Qing Li, Wei-Dong Liu, Lan-Fu Zhang, Daniel Falush, Yang Zhang, Kai-Feng Pan

**Affiliations:** 1 State Key Laboratory of Holistic Integrative Management of Gastrointestinal Cancers, Beijing Key Laboratory of Carcinogenesis and Translational Research, Department of Cancer Epidemiology, Peking University International Cancer Institute 713853, Beijing, China; 2 The Center for Microbes, Development and Health, CAS Key Laboratory of Molecular Virology and Immunology, Institute of Immunity and Infection,Chinese Academy of Sciences 85402 , Shanghai, China; 3 National Key Laboratory of Intelligent Tracking and Forecasting for Infectious Diseases,National Institute for Communicable Disease Control and Prevention,Chinese Center for Disease Control and Prevention 96698, Beijing, China; 4 Health Bureau of Linqu County, Weifang, China; 5 Key Laboratory of Carcinogenesis and Translational Research (Ministry of Education/Beijing), Department of Cancer Epidemiology,Peking University Cancer Hospital & Institute 12519, Beijing, China; Cleveland Clinic, Cleveland, Ohio, USA

**Keywords:** *Helicobacter pylori*, antibiotic resistance, gastric cancer, whole-genome sequencing

## Abstract

The increasing antibiotic resistance of *Helicobacter pylori* has had a serious impact on gastric cancer prevention. Our study aimed to profile the genomic characteristics and explore variants associated with resistance in *H. pylori* strains from a high-risk area of gastric cancer in China. We isolated 153 strains from a community-based cohort and assessed their susceptibility to six antibiotics by MIC Test Strip and genomic characteristics by whole-genome sequencing. Phylogenetic analysis identified the strains as an independent cluster within *H. pylori* East Asian population (hpEastAsia). *HefA*, an efflux pump gene, showed the highest differentiation in the Linqu strains compared with the other Chinese strains. Bacterial genome-wide association study (GWAS) identified 86 resistance variants covering 44 genes. Novel resistance variants were found in *lon* and *babA* for metronidazole, *HP1168* for clarithromycin, *hcpC* for levofloxacin, and *sabA* for rifamycin. Two newly identified *hefA* mutations (R229K and A283V) showed significant associations with metronidazole (*P* = 0.012) and tetracycline (*P* = 0.044) resistance, respectively. *HefA* mutations and GWAS variants were integrated with the significant literature-reported mutations to optimize the prediction models for metronidazole, levofloxacin, clarithromycin, and tetracycline resistance with area under the receiver operating characteristic curves of 0.82–0.93. Double-antibiotic resistance models were established for clinical applicability. Furthermore, *hefA* expression may play a potential mediating role in the associations between mutations and resistance. This study identified genetic independence in the representative *H. pylori* strains from a high-risk area of gastric cancer. Optimized resistance prediction panels, including novel *hefA* mutations and GWAS variants, may provide preliminary guidance for localized precise treatment and helpful experiences for the similar high-risk populations.

**IMPORTANCE:**

*Helicobacter pylori* is a remarkable pathogen due to its virulence in gastric cancer and high genetic plasticity. Linqu County in China, a high-risk area of gastric cancer, faces serious antibiotic resistance issues and necessitates genomic profiling of local *H. pylori* strains. Phylogenetic analysis revealed the Linqu strains as a relatively independent cluster within the hpEastAsia population. Novel antibiotic resistance-associated *hefA* mutations and variants from our bacterial genome-wide association study in the Linqu strains were optimized to improve the prediction performances for single antibiotic and double-drug combination resistance compared with traditional literature-reported mutations. This study identified relative genetic independence and high differentiation in the representative *H. pylori* strains from a population with high risk of gastric cancer and high prevalence of antibiotic resistance. The optimized panels with novel variants improve antibiotic resistance prediction models compared with literature-reported mutations, providing guidance for localized precise treatment and suggesting prevention strategies for similar high-risk populations.

## INTRODUCTION

*Helicobacter pylori* is a remarkable pathogen due to its high virulence in gastric cancer and high genomic plasticity. Extensive geographic variations in *H. pylori* pathogenicity can be attributed to the diverse genome shaped by selection pressures from host global migration and nutritional adaptation (1), such as the high-risk strains of East Asia (2). Linqu County is a distinct region in China with a high mortality rate of gastric cancer (age-adjusted rates per 100,000 were 55 for men and 19 for women) (3) and high prevalence of *H. pylori* infection (4). Our previous interventions in Linqu demonstrated that *H. pylori* eradication is an effective gastric cancer prevention strategy (5). Nevertheless, serious challenges have emerged, with 27% of active treatments failing in our large-scale trial (6), in which antibiotic resistance may be one of the reasons. The genomic characteristics of the *H. pylori* strains still require further investigations in the high-risk area on resistance profiles and underlying mechanisms for precise treatment.

Genetic mutation is one of the major mechanisms for *H. pylori* antibiotic resistance. Important mutations have been reported, including A2142G and A2143G in *23 ribosomal subunit (23S rRNA)* that inhibit the binding of clarithromycin, the substitutions in *16 ribosomal subunit (16S rRNA)* that interfere with the tetracycline function, and the substitutions in *gyrA/B* that alter the target of levofloxacin (7). Upregulated efflux pump systems and the formation of biofilm may also contribute to antibiotic resistance (7). The high genomic plasticity of *H. pylori* under environmental stresses in the high-risk area suggests distinctive resistance mechanisms in the Linqu strains and based on unique variants, rather than relying solely on literature-reported resistance mutations from other areas.

The development of whole-genome sequencing (WGS) has provided a powerful platform to decipher *H. pylori* genotypic characteristics (8). Recent WGS-based studies in different populations confirmed many genotypic and phenotypic resistance correlations, such as *23S rRNA* mutation and clarithromycin, *gyrA/B* mutation and levofloxacin, and revealed novel genetic mechanisms, as well (9). However, hospital-based studies may not adequately represent the local strain characteristics (10, 11). A community-based WGS study, especially in high-risk area, is essential for profiling the representative resistance features and identifying target mutations for localized treatment strategies.

Depending on the community-based cohort in Linqu County, our study systematically investigated the genomic characteristics of the representative *H. pylori* strains and assessed both phenotypic and genotypic profiles of antibiotic resistance. Prediction models were constructed and optimized based on specific variants, which will provide localized, precise prevention strategies for similar high-risk areas.

## MATERIALS AND METHODS

### Participants and samples

Linqu County, a representative rural area in Shandong Province, China, has a population of approximately 820,000 and an area of 1,831 km². This county possesses one of the highest gastric cancer mortality rates worldwide (age-adjusted mortality rates per 100,000 were 55 for men and 19 for women) (3). The National Upper Gastrointestinal Cancer Early Detection Project was launched in Linqu County in 2008 involving annual endoscopic screening of 1,500–3,000 residents aged 40–69 years. For each participant, biopsies were collected from gastric antrum, angle, corpus, and the samples were diagnosed with superficial gastritis, chronic atrophic gastritis, intestinal metaplasia, or dysplasia. From May 2012 to June 2013, we randomly selected two villages and invited eligible project volunteers according to the inclusion criteria (12) to provide one extra fresh biopsy each subject from antrum/angle, which were stored in brain–heart infusion medium for *H. pylori* isolation. In total, we enrolled 468 subjects who provided fresh biopsies and successfully isolated 165 *H*. *pylori* strains. Of these, 153 strains completed antibiotic susceptibility tests and WGS for phenotypic and genotypic profiling (Fig. S1). The characteristics of the participants are shown in Table S1.

### *H. pylori* isolation and antibiotic susceptibility tests

Gastric biopsies were homogenized and inoculated onto nutrient medium containing *H. pylori*-selective supplement (Oxoid Limited, Hampshire, UK). The samples were cultured for 48–72 h in a microaerophilic environment (85% N₂, 10% CO_₂_, 5% O_₂_) at 37℃ and humidity of 95% for *H. pylori* isolation.

Sufficient bacterial growth, indicating optimal growth conditions and an adequate bacterial load, was confirmed to ensure the reliability of antibiotic susceptibility testing. For *H. pylori* strains with scanty growth, additional subculturing was performed to enhance bacterial yield. *H. pylori* strains and the quality control strain ATCC 43504 (provided by the National Institute for Communicable Disease Control and Prevention, Chinese Center for Disease Control and Prevention) were tested for antibiotic susceptibility of amoxicillin, clarithromycin, levofloxacin, metronidazole, rifamycin, and tetracycline using MTS (MIC Test Strip) (Liofilchem, Italy). Campylobacter Agar Base (Karmali, Oxoid Limited, Hampshire, UK) was prepared by dissolving 4.3 g powder in 100 mL sterile deionized water, supplemented with 5% defibrinated sheep blood (Land Bridge Technology, China). Bacterial suspensions were adjusted to a McFarland turbidity of 2.0–2.3 using a densitometer (bioMérieux, France), then uniformly inoculated onto agar plates via sterile swabs. After strip application, the samples were further incubated for 48–72 h until adequate growth was observed. Minimal inhibitory concentration (MIC) values were read according to manufacturer guidelines. The up-to-date EUCAST guidelines (EUCAST Clinical Breakpoint Tables v.13.0, 2023) breakpoints were used to determine resistance.

### DNA extraction and whole-genome sequencing

Genomic DNA of *H. pylori* colonies was extracted using QIAmp DNA Mini Kits (catalog no. 51306). DNA libraries were prepared using NEB Next Ultra DNA Library Prep Kit for Illumina (NEB, USA) and sequenced on the Illumina Novaseq 6000 platform generating 150 bp paired-end reads in FASTQ format. Fastp (v 0.19.7) (13) was used for quality control of the read files. After trimming of reads and adaptors with Trimmomatic (v 0.39) (14), 100× clean reads were extracted by Readfq (v 5, https://github.com/lh3/readfq) for assembly. *De novo* assembly was performed by SPAdes (v 3.13.0) (15) within the Shovill pipeline (v 1.1.0) (https://github.com/tseemann/shovill). The resulting whole-genome assemblies in FASTA format were annotated using Prokka (v 1.14.5) (16).

### Phylogenetic analyses

A total of 1,541 published *H. pylori* strains were obtained from NCBI (https://www.ncbi.nlm.nih.gov/) and Enterobase (https://enterobase.warwick.ac.uk/). We utilized genome-wide haplotype data obtained from the reference-based alignment to *H. pylori 26695* (NC_000915.1). ChromoPainter (v. 0.04) was used to determine the sections of DNA transferred from a donor to a recipient for each recipient haplotype. The results were then consolidated into a “co-ancestry matrix,” which provided information on the number of recombination-derived chunks from each donor to each recipient. In order to cluster individuals based on this matrix, we performed 100,000 iterations of both the burn-in and Markov Chain Monte Carlo (MCMC) chain using fineSTRUCTURE (v. 0.02) (17). To identify region-specific variants of *H. pylori* in Linqu, the fixation index (*Fst*) was calculated on each detected single-nucleotide polymorphism (SNP) site using the R package of PopGenome (18) between Linqu and *H. pylori* East Asian population (hpEastAsia) strains. Additionally, a neighbor-joining phylogenetic tree was constructed using TreeBeST (v1.9.2) (https://github.com/Ensembl/treebest), incorporating 153 Linqu strains and 160 published hpEastAsia strains, including 80 Chinese, 40 Japanese, and 40 Korean strains.

### Genome-wide association study (GWAS)

Snippy (v 4.6.0) (https://github.com/tseemann/snippy) identified the SNPs of the Linqu strains. The unique, overlapping sequence fragments (k-mers) were detected in genome assemblies of the Linqu strains by unitig-caller. The presence or absence of k-mers and SNPs was represented in matrices, as the input files of GWAS. Pyseer (19) was used to fit the fixed models between k-mers, SNPs, and phenotypes of antibiotic resistance. The use of k-mers can identify variants that are not detectable through SNP-based GWAS. The quantile–quantile (Q-Q) plots of GWAS tests based on SNPs and k-mers are shown in Fig. S2 and S3, respectively. To adjust for population structure, we performed multidimensional scaling on the distance matrix of the Linqu strains and retained 10 dimensions when fitting the fixed models. A significance threshold of *P* < 10⁻⁵ (20) was applied to filter SNPs and k-mers associated with resistance phenotypes. The significant k-mers were mapped to *H. pylori 26695* by Nucleotide Basic Local Alignment Search Tool (BLAST) and annotated based on the genome annotation file of *H. pylori 26695*.

### Supplementation of literature-reported resistance mutations

To supplement GWAS, we selected 195 literature-reported resistance mutations across 11 genes (Table S2) associated with six antibiotics from a high-quality review (7) and the Comprehensive Antibiotic Resistance Database (CARD). Sequencing data for candidate resistance genes were subjected to multi-sequence alignment by the MUSCLE program, keeping *H. pylori 26695* as the reference genome. Candidate mutations were evaluated in the Linqu strains by comparing their presence with the corresponding antibiotic resistance rates using the *χ^2^* test or Fisher’s exact test and with the minimum inhibitory concentration (MIC) values using the Mann–Whitney test.

### Construction of prediction models for antibiotic resistance

We integrated novel k-mers and SNPs from GWAS, *hefA* mutations, and literature-reported mutations to construct resistance prediction models. The 153 Linqu strains were randomly divided into a training set for model construction and a test set for validation at a 7:3 ratio. Logistic regression was performed using the “lrm” function in the R package “rms” with a stepwise method to filter variables. The performance of the models was assessed using receiver operating characteristic (ROC) curve analysis by the “pROC” and “ggplot2” packages (v3.1.0).

### Reverse transcription–qPCR (RT-qPCR) and *hefA* gene expression analysis

Total mRNA was extracted from 49 Linqu strains with different antibiotic susceptibility profiles using TRIzol reagent (Tiangen Biotech, China). The reverse transcription was performed using the FastKing cDNA reagent kit (Tiangen Biotech, Beijing, China) according to the manufacturer’s instructions. The expression level of *hefA* was quantified by RT-qPCR, with the *16S rRNA* gene as an internal reference using QuantStudio (TM) 6 Flex System and TB Green Premix Ex Taq II (Takara Bio, Kyoto, Japan). The primers for *hefA* (21) were F (5′-TATGCCCGCTGTTGA-3′) and R (5′-TATGCCCGCTGTTGA-3′). The primers of *16S rRNA* were F (5’- AGACACGGTCCAGACTCCTA-3′) and R (5’- ATCTAATCCTGTTTGCTCCC-3′). Comparison and graphing of expression levels between groups were completed using GraphPad Prism (v 9.0). Mediation models were used to explore the role of *hefA* expression in the association between mutations and resistance by the “mediation” package in R (v 4.5.0), with statistical significance assessed via 5,000 bootstrapped iterations.

## RESULTS

### The resistance phenotypes of the Linqu strains

**Fig 1:**
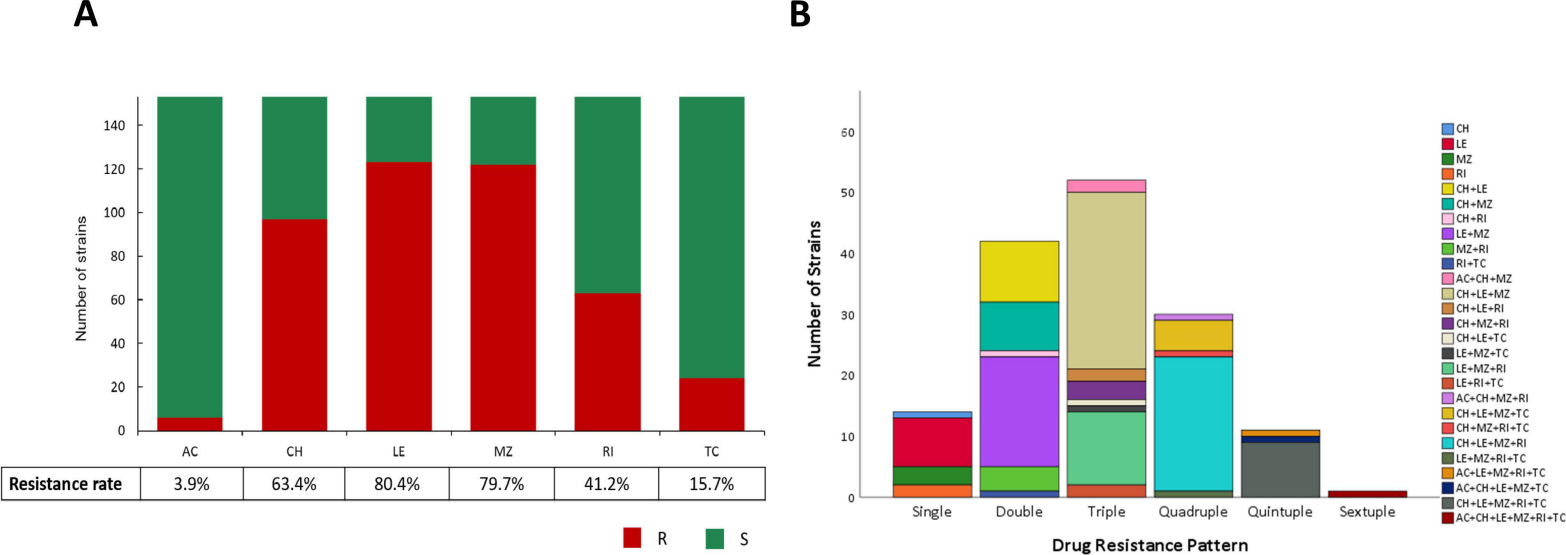
Antibiotic resistance profiling of the Linqu strains. (**A**) The resistance rates in the Linqu strains to AC, CH, LE, MZ, TC, and RI. (**B**) The antibiotic resistance patterns in the Linqu strains, describing the numbers of resistant strains and antibiotic combinations in the single- and multiple-drug resistance groups. AC, amoxicillin; CH, clarithromycin; LE, levofloxacin; MZ, metronidazole; RI, rifamycin; TC, tetracycline; R, resistant; S, susceptible.

Among the 165 *H*. *pylori* strains from Linqu, 153 (92.7%) had resistance phenotypes determined by the MTS test according to EUCAST guidelines (EUCAST Clinical Breakpoint Tables v.13.0, 2023). The distribution of gastric lesions was as follows: 73.9% superficial gastritis, 17.6% chronic atrophic gastritis, 6.5% intestinal metaplasia, and 1.3% dysplasia. The phenotypic resistance frequencies of the Linqu strains were 80.4% for levofloxacin, 79.7% for metronidazole, 63.4% for clarithromycin, 41.2% for rifamycin, 15.7% for tetracycline, and 3.9% for amoxicillin, as determined by the MTS test (Fig. 1A). Only 14 strains (9.2%) exhibited single-drug resistance, affecting levofloxacin, metronidazole, rifamycin, or clarithromycin. Most strains showed resistance to double (27.5%), triple (34.0%), or quadruple (19.6%) antibiotics, with the most common resistance combinations being clarithromycin +levofloxacin + metronidazole (19.0%), clarithromycin + levofloxacin + metronidazole + rifamycin (14.4%), and levofloxacin + metronidazole (11.8%). Simultaneous resistance to five antibiotics was observed in 11 strains (7.2%) (Fig. 1B).

### Population structure

**Fig 2:**
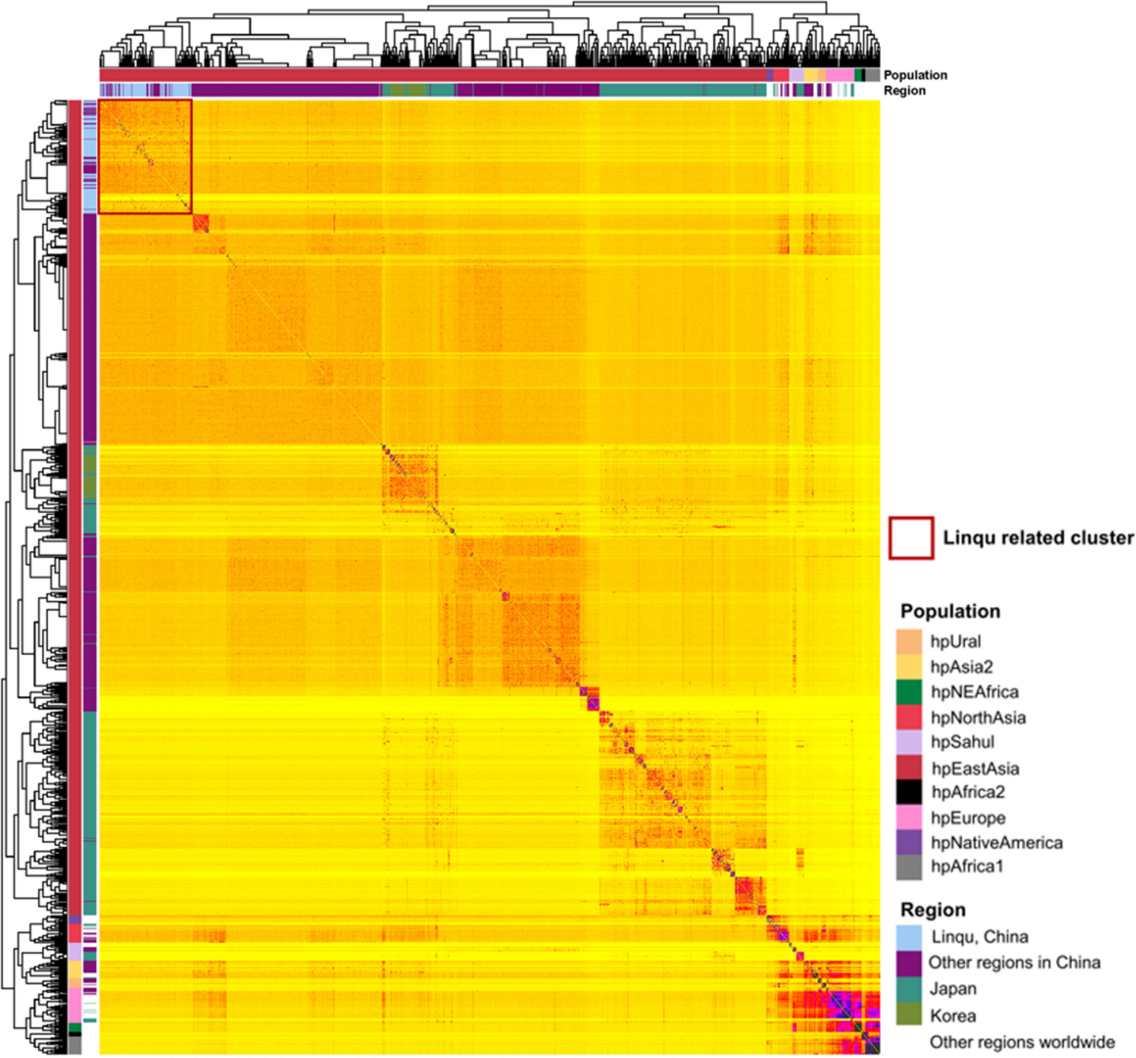
Population structure of Linqu strains and global published strains co-ancestry matrix was calculated using fineSTRUCTURE, including 153 Linqu strains and 1541 published strains with 824 from other regions of China, 494 from Japan, 71 from Korea, and 152 from other continents. The columns on the top and left side show the different geographic regions and populations of the *H. pylori* strains with distinct colors.

Given the high risk of gastric cancer and high antibiotic resistance in Linqu, a phylogenetic assessment was conducted to compare the representative strains with 1,541 published strains, including 824 from other regions of China, 494 from Japan, 71 from Korea, and 152 from other continents. The Linqu strains were classified into hpEastAsia population and formed a distinct Linqu-related cluster with only a few strains from other regions of China (Fig. 2). The phylogenetic tree showed that the Linqu strains were genetically closely related, with various gastric lesions distributed evenly throughout the strains (Fig. S4).

To identify highly differentiated SNPs, we selected those with the highest *Fst* values in the Linqu strains compared with the other hpEastAsia strains. These SNPs were located in *HP1512* (encoding TonB-dependent receptor), *HP0971* (encoding TolC family protein), and *HP1167* (*hofH*, encoding outer membrane beta-barrel protein HofH). Further significant SNPs were found in the Linqu strains compared with the other Chinese hpEastAsia strains on *HP0605* (*hefA*, encoding efflux RND transporter outer membrane subunit HefA), *HP0913* (*alpB*, encoding Hop family adhesin AlpB), and *HP0486* (*hofC*, encoding outer membrane beta-barrel protein HofC). A highly differentiated site in *hefA* (*Fst* > 0.6) was still observed between the Linqu strains and the other strains in the Linqu-related cluster (Fig. S5).

### Resistance-associated variants by GWAS

**Fig 3:**
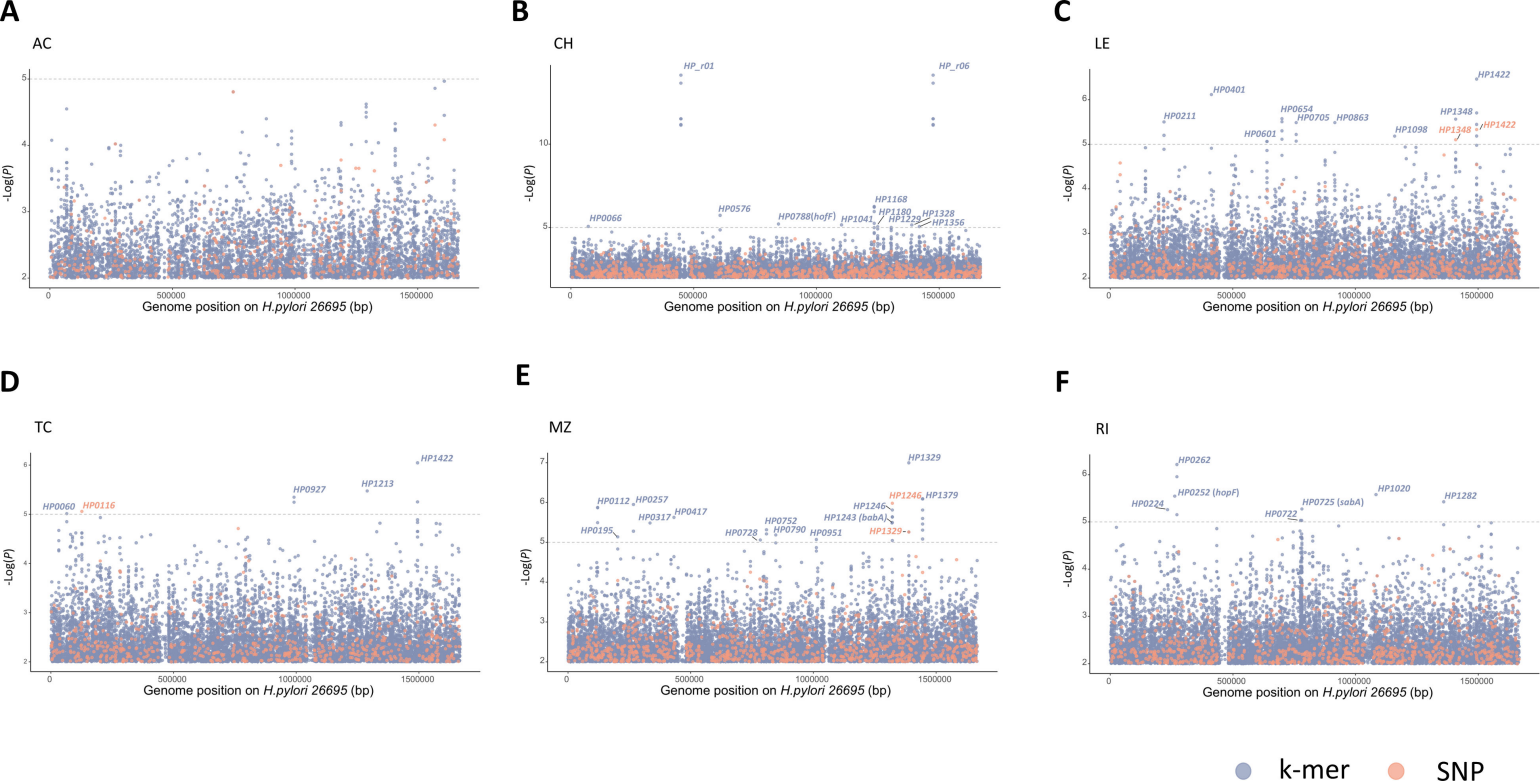
Manhattan plots of the genome-wide association study for antibiotic resistance. Significant antibiotic resistance-associated genetic variations were selected by GWAS between the sensitive and resistant strains for (**A**) AC, (**B**) CH, (**C**) LE, (**D**) TC, (**E**) MZ, and (**F**) RI. Log_10_ (***P***) for each hit is recorded on the vertical axis. The gray dashed line indicates *P* = 10^−5^. GWAS, genome-wide association study; AC, amoxicillin; CH, clarithromycin; LE, levofloxacin; TC, tetracycline; MZ, metronidazole; RI, rifamycin.

Our GWAS systematically investigated resistance variants in the Linqu strains and identified 86 significant variants (81 k-mers and 5 SNPs) associated with clarithromycin, levofloxacin, metronidazole, rifamycin, and tetracycline resistance (Table S3). No resistance variant was found for amoxicillin (Fig. 3A). The most significant variants for clarithromycin resistance were located within the repetitive *23S rRNA* genes (*HP_r01* and *HP_r06*) (Fig. 3B) and consistent with the literature-reported A2143G. *HP1422* (*ileS*), encoding isoleucine-tRNA ligase, was hit by the most significant variants for both levofloxacin (Fig. 3C) and tetracycline resistance (Fig. 3D). Several outer-membrane protein (OMP) genes were implicated in resistance to clarithromycin, including *HP0788* (*hofF*) (Fig. 3B), to metronidazole, including *HP0317* and *HP1243* (*babA*) (Fig. 3E), to rifamycin, including *HP0252* (*hopF*), *HP0722,* and *HP0725* (*sabA*) (Fig. 3F). Our GWAS identified several novel resistance genes, such as *HP1379* (*lon*) for metronidazole, *HP1168* for clarithromycin, and *HP0211* (*hcpA*) for levofloxacin.

As a specifically differentiated efflux pump gene in the Linqu strains, *hefA* sequence was multi-aligned with the reference *H. pylori 26695* and identified nine mutations with frequencies ranging from 5% to 95% (Table S4). The substitution of arginine (R) with lysine (K) at codon 229 (R229K) was significantly associated with an increased MIC median for metronidazole from 4.00 μg/mL to 257.00 μg/mL (*P* = 0.012). The mutation from alanine (A) to valine (V) at codon 283 (A283V) was associated with an increased MIC median for tetracycline from 0.25 to 0.50 (*P* = 0.044).

To supplement GWAS, 195 candidate literature-reported mutations (Table S2) were detected in the Linqu strains, with 29 showing frequencies of 5%–95% (Table S5). Higher MICs and resistance frequencies were identified for mutant K464_D465insE/K/D and T593A/G/K/*P*/S in *pbp1* to amoxicillin, A2143G in *23S rRNA* to clarithromycin, N87K/R and D91N/Y/G in *gyrA* to levofloxacin, A926C/G/T and A928C in *16S rRNA* to tetracycline (all *P* < 0.05). Three candidate mutations with higher MICs were also identified, including the mutant A68G/H/M/R/S/T/V in *rdxA* or R106G/K/L in *frxA* to metronidazole (*P* = 0.040 and 0.030) and the mutant G595S in *pbp1* to amoxicillin (*P* < 0.001) (Table 1).

**TABLE 1:** Significant resistance mutations on literature-reported genes in the Linqu^*a*^

Antibiotics	Resistance genes	Mutation sites	Genotype	Resistance phenotype (%)		P	MIC median (interquartile, μg/mL)	*P* ^*f*^
				Susceptible	Resistant			
AC	*pbp1*	K464_D465insE/K/D^*b*^	W	142 (97.93)	3 (2.07)	**0.001** ^*e*^	0.015 (0.015, 0.023)	**<0.001**
			M	4 (57.14)	3 (42.86)		0.125 (0.047, 0.250)	
		T593A/G/K/*P*/S^*b*^	W	124 (98.41)	2 (1.59)	**0.009** ^*e*^	0.015 (0.015, 0.016)	**<0.001**
			M	23 (85.19)	4 (14.81)		0.032 (0.016, 0.072)	
		G595S^*b*^	W	92 (97.87)	2 (2.13)	0.21^*e*^	0.015 (0.015, 0.016)	**<0.001**
			M	55 (93.22)	4 (6.78)		0.016 (0.015, 0.032)	
CH	*23S rRNA*	A2143G^*c*^	W	54 (59.34)	37 (40.66)	**<0.001** ^*d*^	0.19 (0.13, 0.50)	**<0.001**
			M	2 (3.64)	53 (96.36)		24.00 (16.00, 64.00)	
LE	*gyrA*	N87K/R^*b*^	W	28 (22.58)	96 (77.42)	**0.05** ^*e*^	2.50 (1.50, 33.00)	**<0.001**
			M	1 (4.00)	24 (96.00)		33.00 (33.00, 33.000)	
		D91N/Y/G^*b*^	W	29 (24.37)	90 (75.63)	**0.003** ^*e*^	2.00 (1.50, 33.00)	**<0.001**
			M	0 (0.00)	30 (100.00)		33.00 (33.00, 33.00)	
MZ	*rdxA*	A68G/H/M/R/S/T/V^*b*^	W	27 (22.31)	94 (77.69)	0.19^*d*^	257.00 (12.00, 257.00)	**0.04**
			M	3 (11.11)	24 (88.89)		257.00 (128.00, 257.00)	
	*frxA*	R106G/K/L^*b*^	W	30 (21.28)	111 (78.72)	0.21^*e*^	257.00 (32.00, 257.00)	**0.03**
			M	0 (0.00)	10 (100.00)		257.00 (257.00, 257.00)	
TC	*16S rRNA*	A926C/G/T^*c*^	W	115 (87.79)	16 (12.21)	**0.006** ^*e*^	0.50 (0.25, 0.75)	**0.001**
			M	13 (61.90)	8 (38.10)		1.00 (0.50, 4.50)	
		A928C^*c*^	W	123 (86.62)	19 (13.38)	**0.01** ^*e*^	0.50 (0.25, 0.88)	**<0.001**
			M	5 (50.00)	5 (50.00)		1.50(0.94, 4.25)	

^*a*^ AC, amoxicillin, CH, clarithromycin, LE, levofloxacin, MZ, metronidazole, TC, tetracycline, W, wild type, M, Mutant type, MIC, minimum inhibitory concentration.

^*b*^ Mutations are defined using *H. pylori 26695* as the reference genome and reported following standard recommendations in molecular diagnostics from the Human Genome Variation Society. For genes coding proteins, mutations in codons were described.

^*c*^ For genes coding RNA, the base positions of the mutations were described.

^*d*^ Chi-square test for the comparison of resistance rate between wild- and mutant-type strains. Bold values indicate statistically significant results (*P* < 0.05).

^*e*^ Fisher’s exact test for the comparison of resistance rate between wild and mutant type strains. Bold values indicate statistically significant results (*P* < 0.05).

^*f*^ Man-Whitney test for the comparison of MIC between wild and mutant type strains. Bold values indicate statistically significant results (*P* < 0.05).

### Antibiotic resistance prediction models

**Fig 4:**
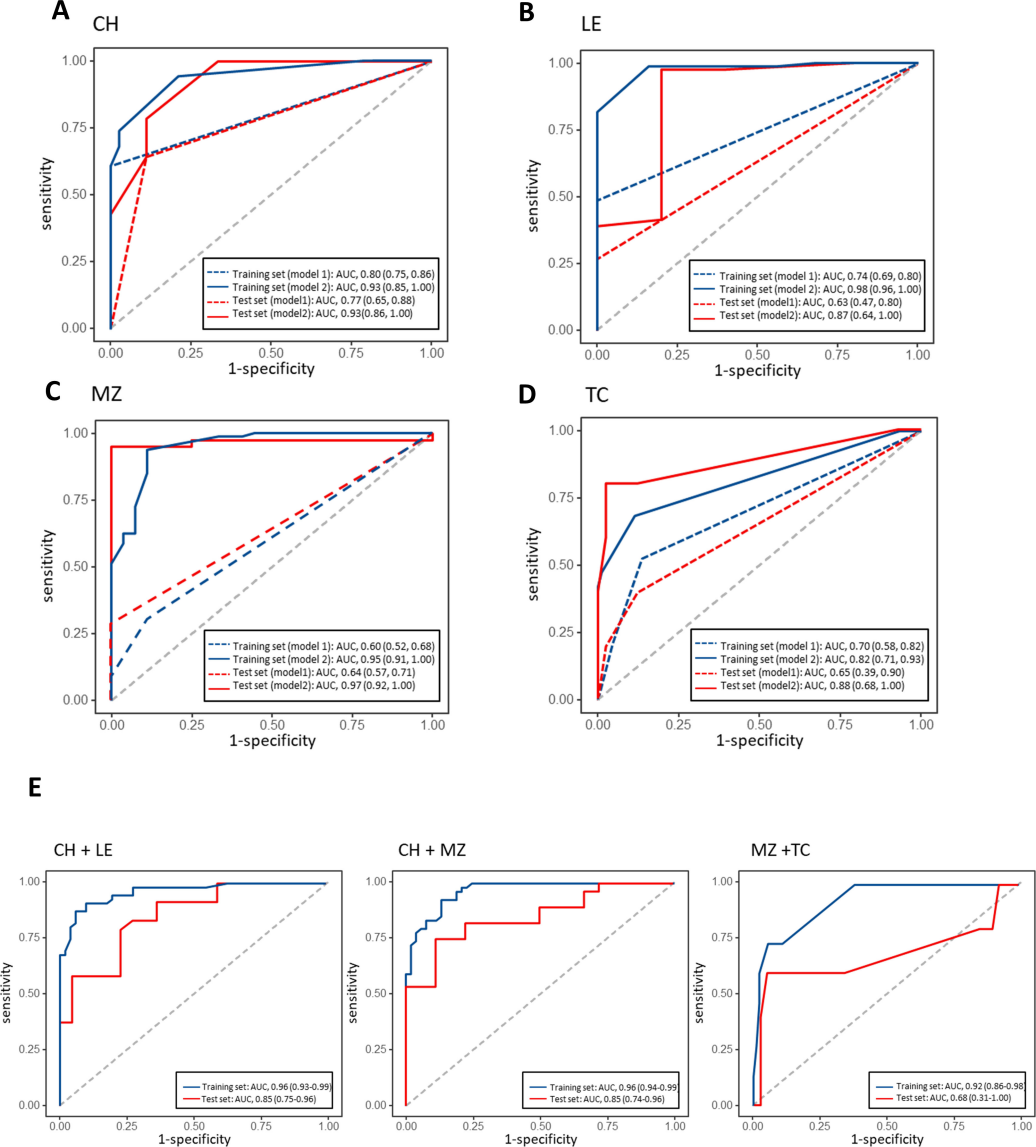
The resistance prediction model construction and validation in the Linqu strains. Prediction models were constructed and validated for single and double-drug resistance, including (**A**) CH, (**B**) LE, (**C**) MZ, (**D**) TC, (**E**) CH + LE, CH + MZ, MZ + TC. ROC curve analyses were conducted to evaluate the prediction models including model 1 (literature-reported mutation panel) and model 2 (optimized panel combining literature-reported mutations, *hefA* mutations, and novel GWAS variants). The model uses genetic data as input to predict susceptible or resistant phenotypes as output. GWAS, genome-wide association study; CH, clarithromycin; LE, levofloxacin; MZ, metronidazole; TC, tetracycline; ROC, receiver operating characteristic.

Using significant variants, we constructed and validated resistance prediction models in training and test sets from the Linqu strains (Table 2). Model 1 was constructed using only literature-reported mutations for clarithromycin, levofloxacin, metronidazole, tetracycline, and amoxicillin with area under the curve (AUCs) values of ROCs of 0.80 (0.75–0.86), 0.74 (0.69–0.80), 0.60 (0.52–0.68), 0.70 (0.58–0.82), and 0.99 (0.97–1.00) in the train set, respectively. These models were validated in the test set, yielding AUCs of 0.77 (0.65–0.88), 0.64 (0.57–0.71), 0.64 (0.57–0.71), 0.65 (0.39–0.90), and 0.61 (0.23–0.99), respectively (Fig. 4Athrough D ; Fig. S6A).

**TABLE 2:** The prediction models for antibiotic resistance^*a*^

Resistance prediction models	Source of genetic variants	Antibiotic resistance genes and mutations
CH		
Model 1	1 literature-reported mutation	*23S rRNA* (A2143G)
Model 2	1 literature-reported mutation	*23S rRNA* (A2143G)
	3 GWAS variants	*flhA*, *HP1168*, *HP1229*
LE		
Model 1	2 literature-reported mutations	*gyrA* (N87K/R, D91N/Y/G)
Model 2	2 literature-reported mutations	*gyrA* (N87K/R, D91N/Y/G)
	5 GWAS variants	*hcpA, aroA, uvrA, pgbB, ileS*
MZ		
Model 1	2 literature-reported mutations	*rdxA* (A68G/H/M/R/S/T/V), *frxA* (R106G/K/L)
Model 2	1 *HeFA* mutation	*hefA* (R229K)
	7 GWAS variants	*HP0112, fabI, tilS, filD, recO, babA, lon*
TC		
Model 1	2 literature-reported mutations	*16S rRNA* (A928C, A926C/G/T)
Model 2	1 literature-reported mutation	*16S rRNA* (A926C/G/T)
	1 *hefA* mutation	*hefA* (A283V)
	3 GWAS variants	*topA*, *htpX*, *HP1213*
AC		
Model 1	3 literature-reported mutations	*pbp1* (K464_D465ins, N562D/H/Y, T593A/G/K/*P*/S)
RI		
Model 2	4 GWAS variants	*HP0262*, *sabA*, *HP1020*, *trpE*
Double-drug model		
CH +MZ	1 literature reported mutation	*23S rRNA* (A2143G)
	1 *hefA* mutation	*hefA* (R229K)
	9 GWAS variants	*ftsK*, *HP0112*, *fabI*, *metG*, *hofF*, *recO*, *HP1229*, *nadA*, *lon*
MZ +TC	1 literature-reported mutation	*16S rRNA* (A926C/G/T)
	2 *hefA* mutations	*hefA* (A283V, R229K)
	3 GWAS variants	*topA, HP0317, htpX*
CH +LE	2 literature-reported mutations	*23S rRNA* (A2143G), *gyrA* (D91N/Y/G)
	7 GWAS variants	*hcpA, aroA, uvrA, HP1168, HP1229, nadA, ileS*

^*a*^ AC, amoxicillin, CH, clarithromycin; LE, levofloxacin; MZ, metronidazole; RI, rifamycin; TC, tetracycline.

To improve prediction performance, we optimized the panels in model 2 by adding the novel *hefA* mutations and GWAS variants (Table 2). For clarithromycin resistance, the addition of three GWAS variants enhanced the performances with AUCs of 0.93 (0.85–1.00) and 0.93 (0.86–1.00) in the train and test sets (Fig. 4A). Similar improvement was found for levofloxacin resistance by adding five GWAS variants with AUCs of 0.98 (0.96–1.00) and 0.87 (0.64–1.00) in the train and test sets (Fig. 4B). The replacement of literature-reported mutations by *hefA* mutation (R229K) and seven GWAS variants showed superior prediction for metronidazole resistance with AUCs of 0.95 (0.91–1.00) and 0.97 (0.92–1.00) in the train and test sets (Fig. 4C). The tetracycline resistance model combined one literature-reported mutation, one *hefA* mutation (A283V), and three GWAS variants with better AUCs of 0.82 (0.71–0.93) and 0.88 (0.68–1.00) in the train and test sets (Fig. 4D). For rifamycin resistance, we only constructed model 2 using four novel GWAS variants in the train and test sets with AUCs of 0.84 (0.76–0.91) and 0.84 (0.73–0.95) (Fig. S6B).

According to the treatment guidelines (22), we constructed double-drug resistance prediction models for clarithromycin + levofloxacin, clarithromycin + metronidazole and metronidazole + tetracycline. The models optimized the novel and literature-reported variants to distinguish the combined phenotypes with AUCs of 0.96 (0.93–0.99), 0.96 (0.94–0.99), and 0.92 (0.86–0.98) in the training set, 0.85 (0.75–0.96), 0.85 (0.74–0.96), and 0.68 (0.31–1.00) in the test set (Fig. 4E). The panels for the double-drug models were more streamlined, consisting of 11 variants for clarithromycin + metronidazole, six variants for metronidazole + tetracycline, and nine variants for clarithromycin +levofloxacin (Table 2).

### Potential associations between *hefA* and antibiotic resistance

**Fig 5:**
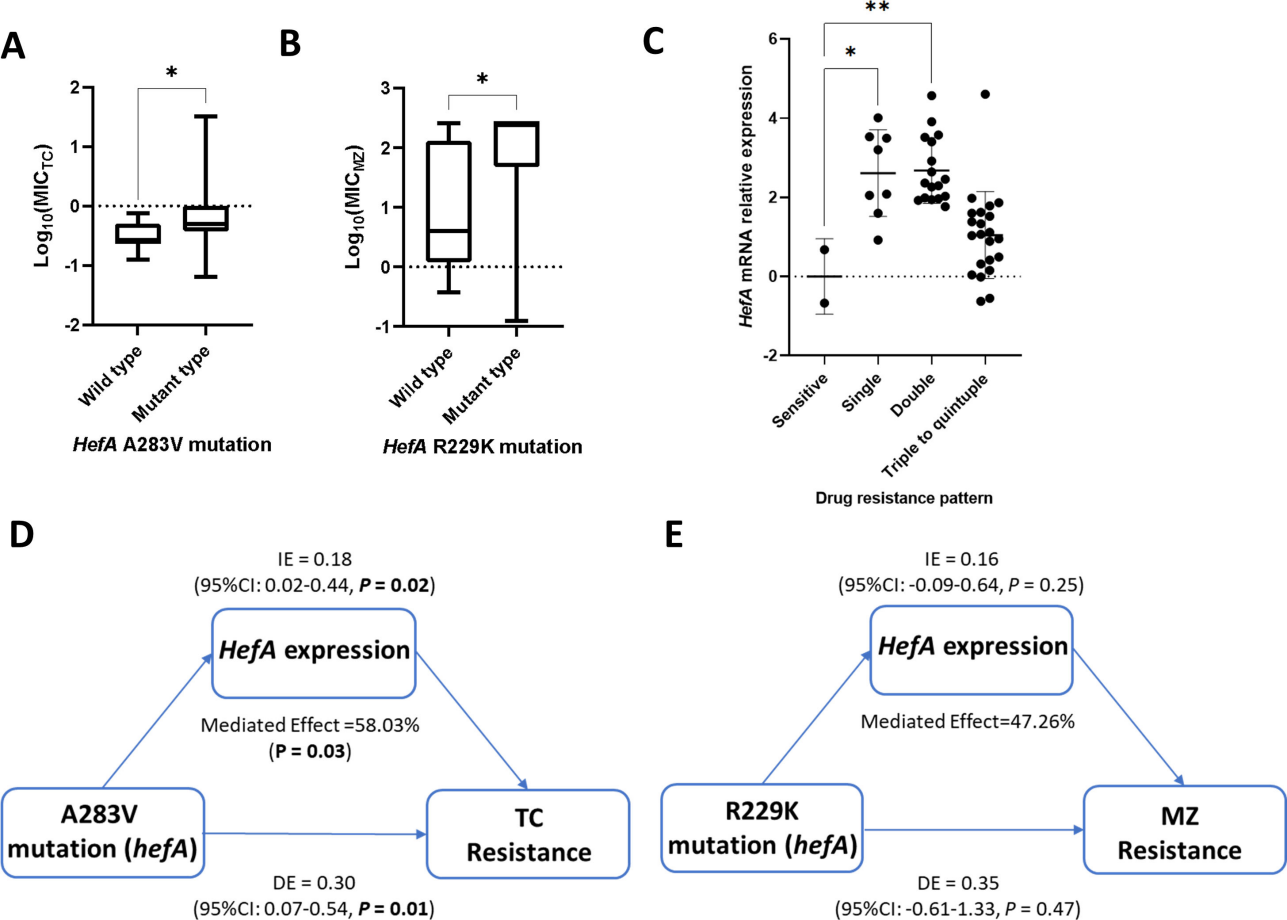
Associations among *hefA* mutations, expression, and antibiotic resistance. (**A**) Association between A283V mutation and the MIC values for TC. (**B**) Association between R229K mutation and the MIC values for MZ. (**C**) Association between *hefA* expression and antibiotic resistance patterns. (**D**) Mediation analysis for the involvement of *hefA* expression in the association between A283V mutation and TC resistance. Mediated effect: percentage of the total effect of *hefA* variants on antibiotic resistance mediated by *hefA* expression; IE: indirect effect; DE: direct effect. (**E**) Mediation analysis for the involvement of *hefA* expression in the association between R229K mutation and MZ resistance. MIC, minimum inhibitory concentration; TC, tetracycline; MZ, metronidazole.

*HefA* was evaluated with antibiotic resistance for the specific differentiation in the Linqu strains and key roles in many prediction models. We identified two significant mutations of A283V with MIC for tetracycline (Fig. 5A) and R229K with MIC for metronidazole (Fig. 5B). However, it is challenging to directly evaluate *hefA* expression with mutations or single resistance phenotypes for metronidazole and tetracycline due to the high mutant frequencies (both 94.1% for R229K and A283V) and the complex multi-drug resistance patterns (88.9% with 23 different resistance combinations) in the Linqu strains. Then, we selected 8 single-drug, 17 double-drug, 22 triple to quintuple-drug resistant strains, and 2 susceptible strains for mRNA evaluation. Compared with the susceptible strains, we found significant increases in *hefA* expression in single-drug (*P* = 0.02) and double-drug (*P* < 0.001) resistant strains with a similar trend in triple to quintuple-drug-resistant strains (*P* = 0.21) (Fig. 5C). Mediation analysis suggested that *hefA* expression significantly contributed to the association between the A283V mutation and tetracycline resistance, mediating 58.03% of the effect (*P* = 0.03) (Fig. 5D). Similarly, *hefA* expression may also play a potential role in mediating the association between the R229K mutation and metronidazole resistance with a mediation proportion of 47.26%, although the *P*-value showed no significance (Fig. 5E).

## DISCUSSION

*H. pylori* eradication is an effective strategy for preventing gastric cancer. The rapid increase in antibiotic resistance poses a serious threat in high-risk areas of gastric cancer, such as Linqu County, China. Understanding the local resistance spectrum and underlying mechanisms is crucial for the precise prevention of gastric cancer in this area. Our study systematically profiled genomic characteristics of the representative Linqu strains, depending on a community-based cohort. We identified novel resistance variants through GWAS and developed prediction models, providing guidance on the precise selection of antibiotics for anti-*H*. *pylori* treatment and gastric cancer prevention in similar high-risk areas.

Our study preliminarily identified higher resistance rates to common antibiotics (63.4% for clarithromycin, 79.7% for metronidazole, 80.4% for levofloxacin, and 15.7% for tetracycline) in the Linqu strains compared with the average levels in China (23). Although large-scale community-based surveys are still needed in Linqu, we ensured the reliability and accuracy of the antibiotic resistance results by following Clinical and Laboratory Standards Institute (CLSI) guidelines and internal quality control protocols. Similar high antibiotic resistance was also found in other high-risk regions in Western and Northern China with 31.0%–50.2% for clarithromycin, 78.0%–92.0% for metronidazole, and 54.9%–56.0% for levofloxacin (24, 25). The distinctive resistance spectrum and complex multi-drug resistance patterns observed in the Linqu strains suggest the need for systematic genomic investigations.

Linqu strains, from the area with high risk of gastric cancer and high frequencies of antibiotic resistance, were classified within the hpEastAsia population as a relatively genetically independent Linqu-related cluster. The most differentiated genes in the Linqu strains encode OMPs including *hefA*, *alpB*, *hofC*, *hofH,* and *tolC*. These differentiated OMPs are involved in adhesion to epithelial cells, penetration through defense barriers, and evasion of the immune system (26), which suggests clues to the pathogenicity of the Linqu strains.

Among these differentiated OMP genes, *hefA* exhibited the highest level of differentiation in the Linqu strains, even when compared with the other strains in the Linqu-related cluster. Two novel *hefA* mutations (R229K and A283V) were significantly associated with metronidazole (*P* = 0.012) and tetracycline (*P* = 0.044) resistance, providing candidate variants for localized prediction models. Encoding an outer membrane efflux pump protein, *hefA* expression was previously found (27) to be associated with multi-drug resistance, which was also currently confirmed in our study (*P* < 0.001). Our mediation analysis suggested possible involvement of *hefA* expression in the association between A283V mutation and tetracycline resistance. However, larger sample-size validation and comprehensive resistance mechanisms are still required.

Several GWAS studies have investigated *H. pylori* pathogenicity in relation to carcinogenesis (20, 28). However, whole-genome scale investigations are still needed to identify antibiotic resistance-associated variants. Our GWAS is the first to systematically screen for resistance variants in a high-risk population and identified novel elements in addition to the well-known mutation in *23S rRNA* (A2143G). Several OMP genes were newly found for the associations with clarithromycin, metronidazole, and rifamycin resistance, including *hofF*, *babA*, *hopF,* and *sabA*. Although most OMP genes were not reported as resistance associated, *babA* and *sabA* may play a role in *H. pylori* anchoring and persistent infection against elimination. Similar to *hefA*, our GWAS found novel resistance variants in efflux system genes, such as *HP1328* for clarithromycin and *HP1329* for metronidazole resistance. The overexpression of resistance–nodulation–division efflux systems has been reported to contribute to multi-drug resistance by reducing the drug concentrations (29). Our GWAS suggested that novel variants in both OMPs and efflux system proteins may contribute to resistance in the Linqu strains.

To supplement GWAS, we identified 10 significant literature-reported mutations associated with amoxicillin, clarithromycin, metronidazole, levofloxacin, and tetracycline resistance in the Linqu strains with well-characterized functions. For example, the mutations of K464_D465insE/K/D and T593A/G/K/*P*/S in *pbp1* may contribute to amoxicillin resistance by reducing the affinity with the antibiotic. Mutations in *gyrA*, including N87K/R (*P* = 0.05) and D91N/Y/G (*P* = 0.003), may alter the target of levofloxacin by protecting DNA synthesis and transcription. Mutations in *16S rRNA* (A926C/G/T, A928C) may interfere with the binding of tetracycline. Although previous studies reported mutations in *frxA* (R106G/K/L) (30) and *rdxA* (A68G/H/M/R/S/T/V) (31) with metronidazole resistance by altering oxygen-insensitive nitroreductases (7) as well as the mutation in *pbp1* (G595S) (32) with amoxicillin resistance, our study only observed weak associations between these mutations and antibiotic resistance in the Linqu strains. The inclusion of literature-reported mutations supplied substantial variants to construct resistance prediction models.

Previous studies have reported resistance prediction for amoxicillin (33), clarithromycin (34), and levofloxacin (9, 11), with the literature-reported mutations in *pbp1*, *23S rRNA* and *gyrA*. The present study combined the novel *hefA* mutations and GWAS variants (such as variants in *babA* and *sabA*) with the significant literature-reported mutations to optimize panels for resistance prediction models. The localized models can significantly improve the prediction performances in Linqu strains compared with the traditional literature-reported mutations and will provide accurate treatment experiences for similar high-risk populations.

Considering the high frequencies and complex patterns of multi-drug resistance in the Linqu strains, we constructed three comprehensive prediction models for double-drug resistance in accordance with treatment guidelines, including clarithromycin + metronidazole, clarithromycin + levofloxacin, and metronidazole + tetracycline. We streamlined and optimized the individual antibiotic resistance panels to obtain more streamlined and efficient double-drug combination models for clinical applicability. However, larger sample-size validations are still required in high-risk populations for a more practicable prediction of antibiotic resistance and localized guidelines of the treatment regimens.

Our study has several strengths. We systematically described and compared the genomic characteristics of the representative strains from Linqu, a high-risk area of gastric cancer with high antibiotic resistance in China. The phenotypic and genotypic resistance profiles were assessed using the MTS test and WGS to identify novel variants in the Linqu strains. Novel variants and literature-reported mutations were optimized to construct resistance prediction models not only for single antibiotics but also for double-drug combination, which may support the localized precise prevention strategy in this high-risk population. However, our study also has limitations. The modest sample size may limit the assessment of the prediction models. For example, only five resistant strains to both metronidazole and tetracycline in the test set may cause low performance of the double-drug combination model. Larger and independent validations are needed to ensure the practicability of the panels and prediction models. Confirmations are needed in an intervention trial with reference to empirical treatment and actual clinical outcomes. Moreover, the novel variants specifically identified in the Linqu strains require functional exploration to gain a deeper understanding of the underlying mechanisms and to aid the development of non-invasive detection methods, such as stool-based assays. Finally, the microaerophilic environment used in the metronidazole susceptibility test has been shown to potentially affect resistance determination (35), and this will be taken into account in future validations.

In summary, our study identified relative genetic independence and high differentiation in the *H. pylori* strains from a population at high risk for gastric cancer and with high levels of antibiotic resistance. Integrated panels including novel *hefA* mutations and GWAS variants can optimize the resistance prediction models, providing preliminary guidance for localized, precise prevention strategies and offering valuable insights for similar high-risk populations.

## Data Availability

Sequencing data of 153 isolates from Linqu County in this study are available on Zenodo under the accession number 14560813 (doi: 10.5281/zenodo.14560813). All data included in this study are available upon request by contact with the corresponding author.

## SUPPLEMENTAL MATERIAL

Supplemental material jcm.00033-25-s0001.docx Figures S1 to S6; Tables S1 to S5.

ASM does not own the copyrights to Supplemental Material that may be linked to, or accessed through, an article. The authors have granted ASM a non-exclusive, world-wide license to publish the Supplemental Material files. Please contact the corresponding author directly for reuse.
