# Supplementary material for: Population structure of Helicobacter pylori and antibiotic resistance-associated variants in a high-risk area of gastric cancer
Source: J Clin Microbiol. 2025 Apr 11;63(5):e00033-25. doi: 10.1128/jcm.00033-25 (PMC12077087; doi:10.1128/jcm.00033-25)
Supplement: Supplemental material — Figures S1 to S6; Tables S1 to S5. [file jcm.00033-25-s0001.docx]

**Population structure of *Helicobacter pylori* and antibiotic resistance associated variations in a high-risk area of gastric cancer**

**Author(s):** Qiu-Yu Jin^1†^, Roberto C Torres^2^, Chao Yang^2^, Li-Hua He^3^, Zong-Chao Liu^1^, Wen-Qing Li^1^, Wei-Dong Liu^4^, Lan-Fu Zhang^4^, Daniel Falush^2^, Yang Zhang^5*^, Kai-Feng Pan^1*^

**Contents**

[Supplementary Figures 2](#_Toc192770575)

[Supplementary Figure 1 2](#_Toc192770576)

[Supplementary Figure 2 3](#_Toc192770577)

[Supplementary Figure 3 4](#_Toc192770578)

[Supplementary Figure 4 5](#_Toc192770579)

[Supplementary Figure 5 6](#_Toc192770580)

[Supplementary Figure 6 7](#_Toc192770581)

[Supplementary Tables 8](#_Toc192770582)

[Supplementary Table 1 8](#_Toc192770583)

[Supplementary Table 2 9](#_Toc192770584)

[Supplementary Table 3 10](#_Toc192770585)

[Supplementary Table 4 15](#_Toc192770586)

[Supplementary Table 5 19](#_Toc192770587)

# Supplementary Figures

## Supplementary Figure 1 Flow chart for sample collection and data analyses


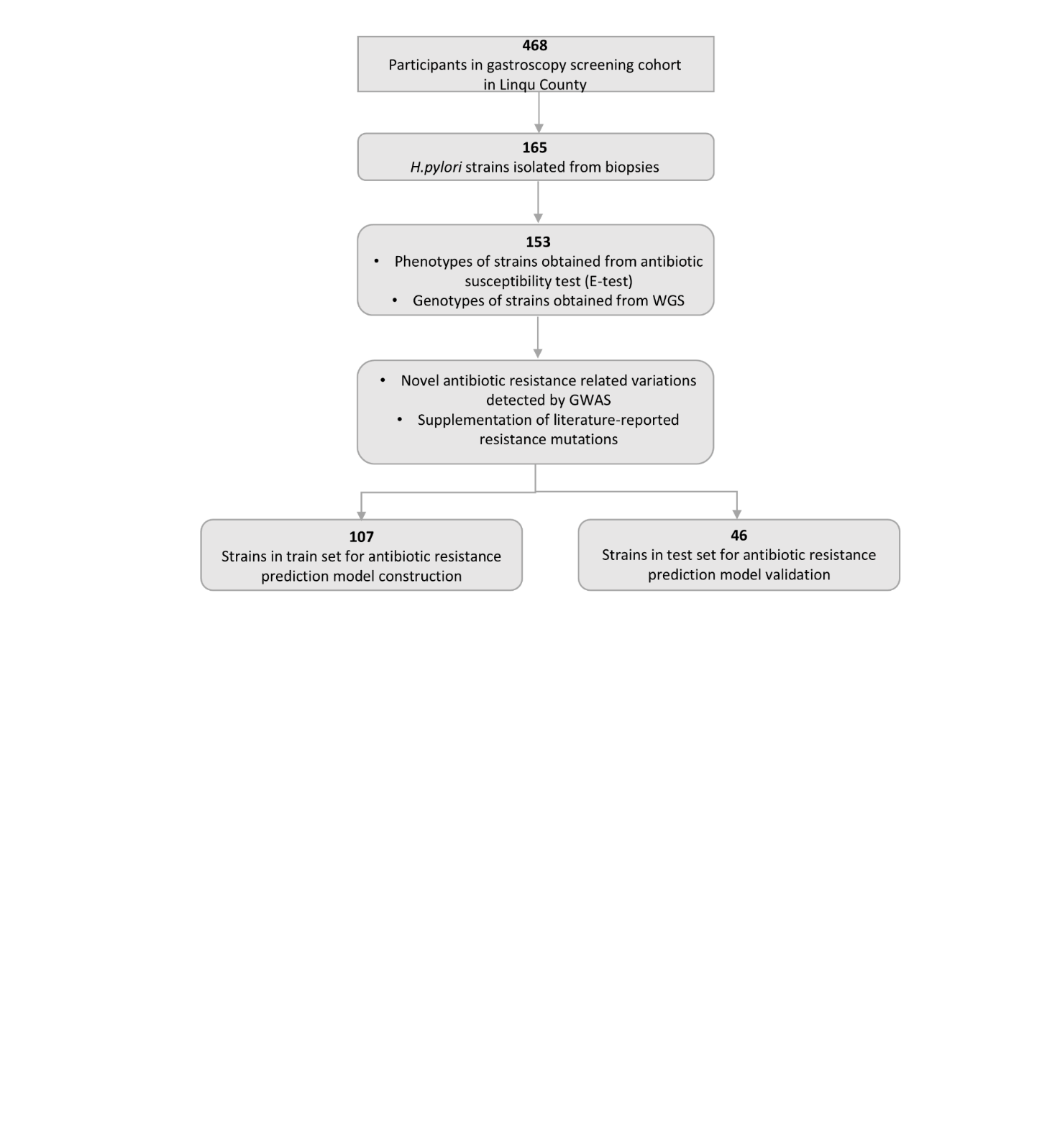


E-test, Epsilometer test; WGS, whole-genome sequencing; GWAS, genome-wide association study

## Supplementary Figure 2 Q-Q plots of GWAS tests between SNPs and antibiotic resistance phenotypes


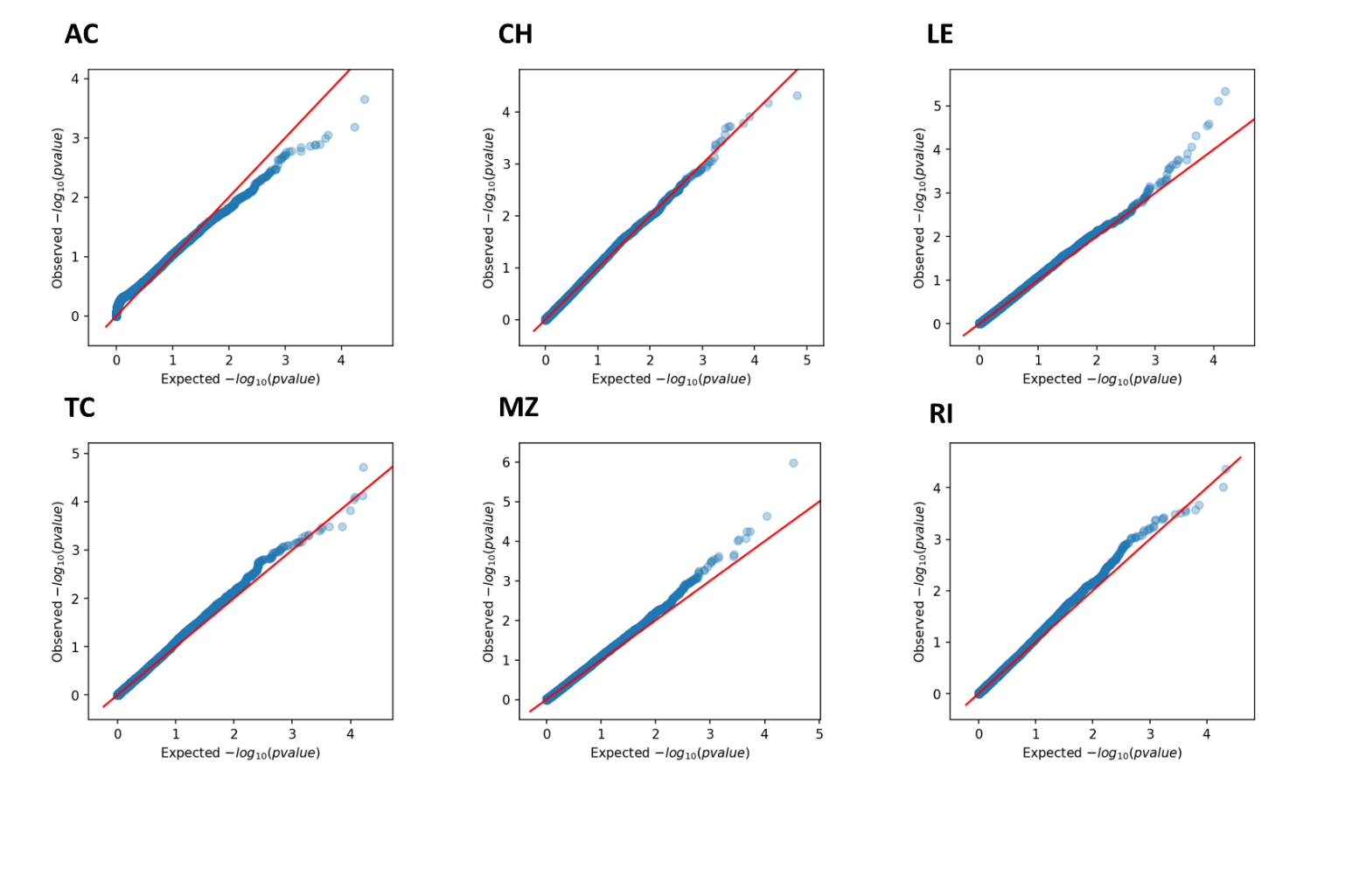


GWAS, genome-wide association study; AC, amoxicillin; CH, clarithromycin; LE, levofloxacin; MZ, metronidazole; RI, rifamycin; TC, tetracycline

## Supplementary Figure 3 Q-Q plots of GWAS tests between k-mers and antibiotic resistance phenotypes


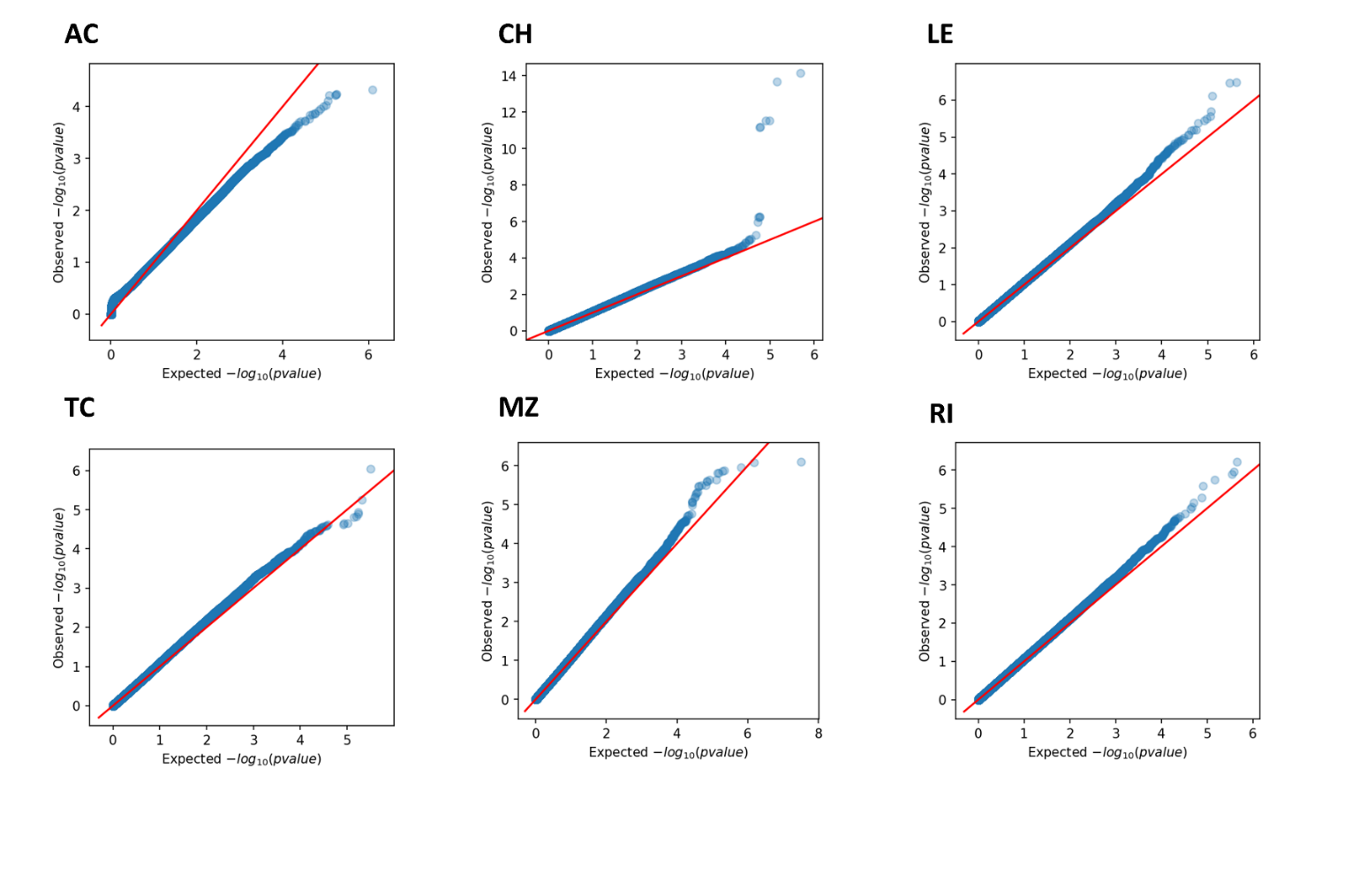


GWAS, genome-wide association study; AC, amoxicillin; CH, clarithromycin; LE, levofloxacin; MZ, metronidazole; RI, rifamycin; TC, tetracycline

## Supplementary Figure 4 Phylogenetic analysis of Linqu strains and hpEastAsia strains


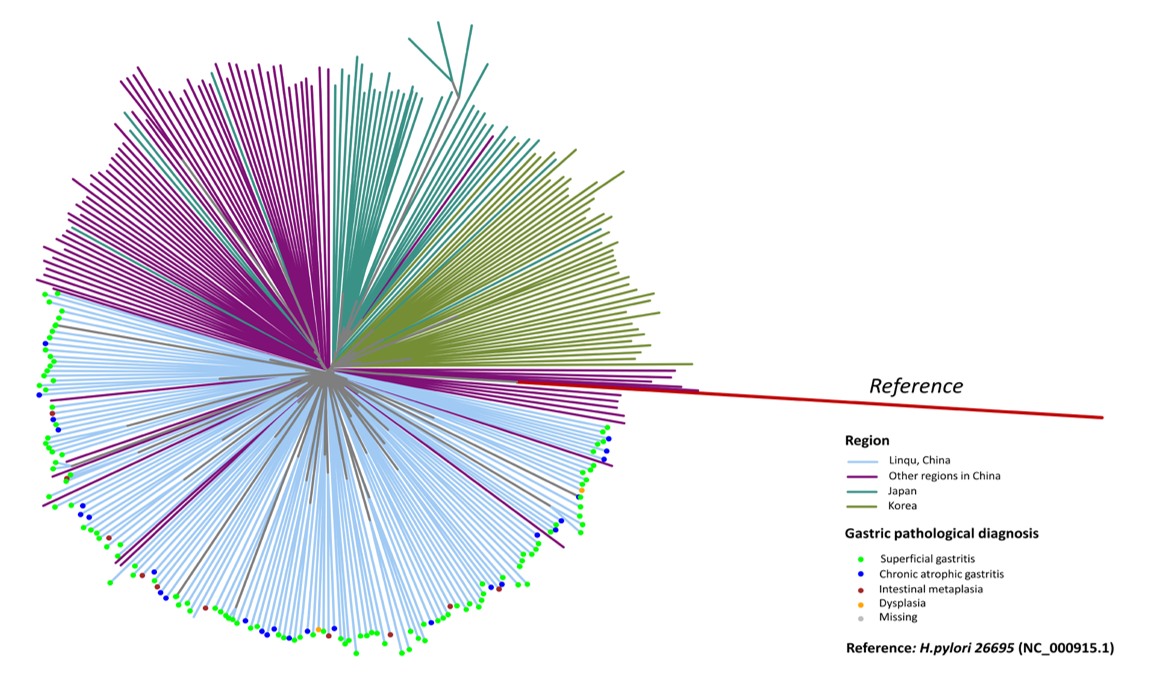


Neighbor-joining phylogenetic tree involving strains from Japan, Korea, Linqu and the other regions of China showed that the Linqu strains were genetically close to each other with various gastric lesions scattering. The color of the branches and the dots represented different geographic regions and different pathological lesions from which the strains were obtained.

## Supplementary Figure 5 Region-specific variants of *H. pylori* in Linqu County


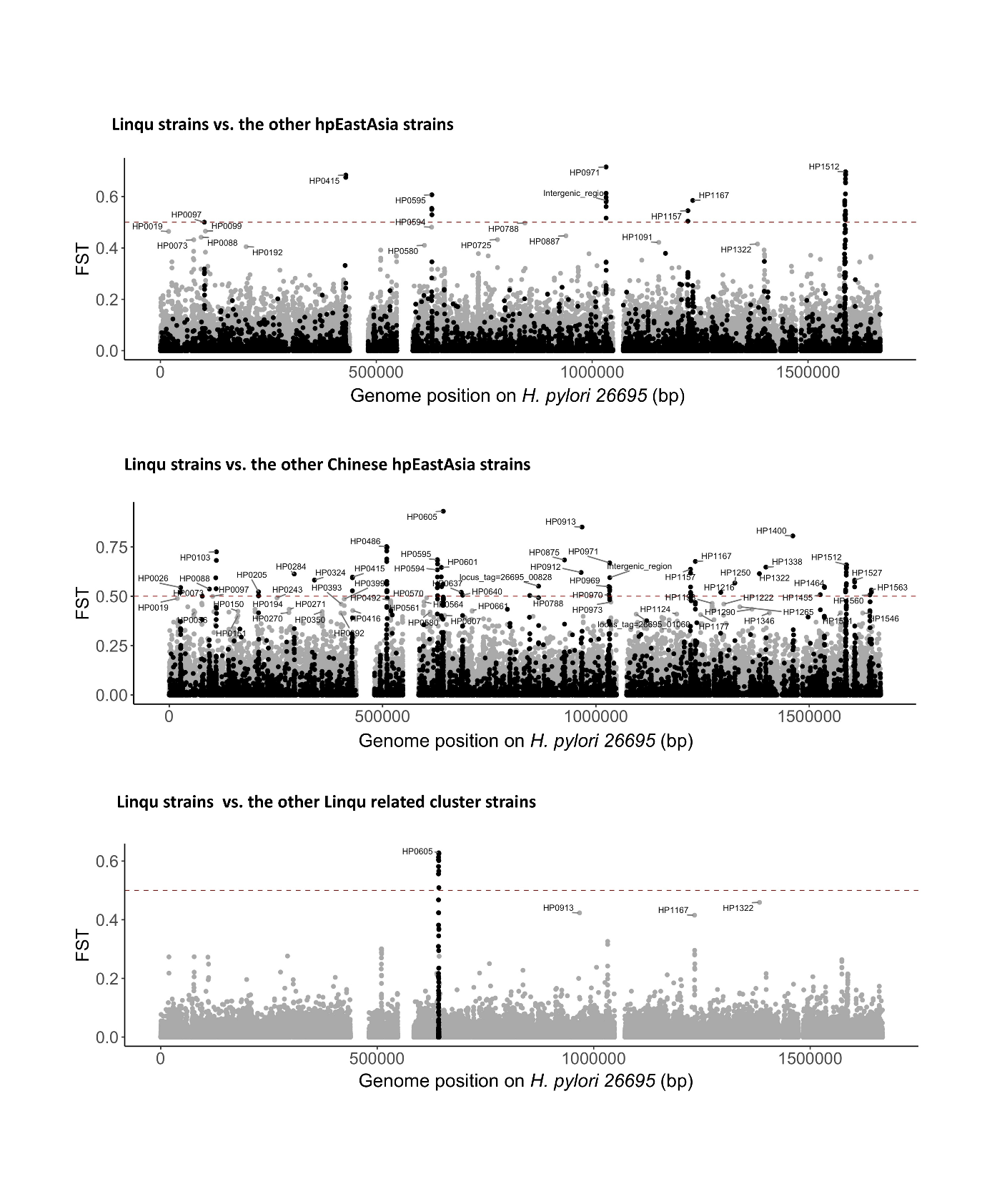


Across the whole-genome of *H. pylori*, the significantly differentiated variations were selected by the comparison of *Fst* values between the Linqu strains and the other hpEastAsia strains, the other Chinese hpEastAsia strains, and the other Linqu related cluster strains.

*Fst*, fixation index.

## Supplementary Figure 6 Performance of the resistance prediction model for amoxicillin and rifamycin


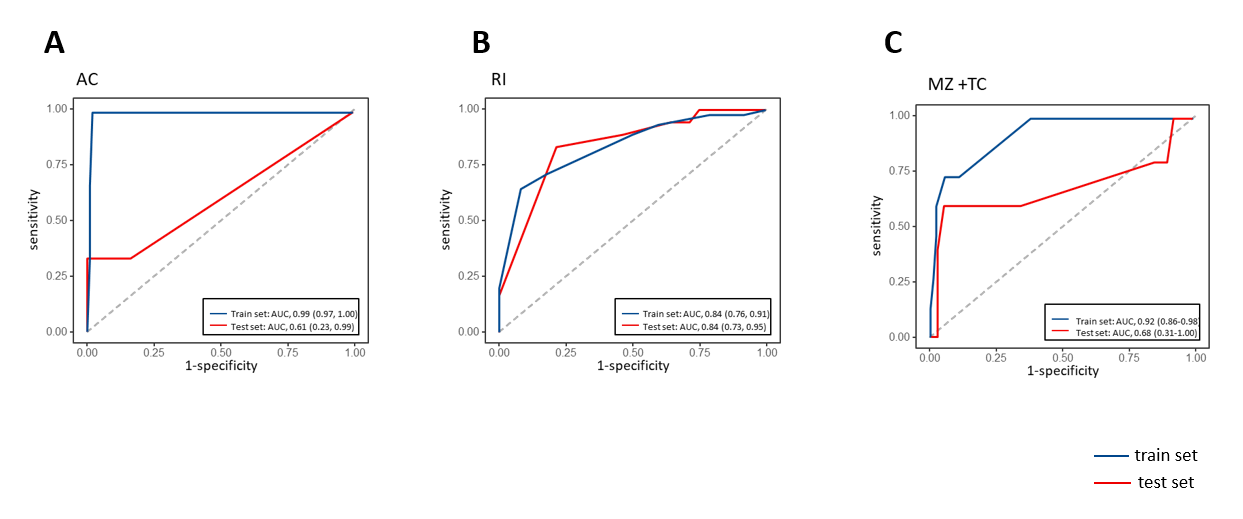


Receiver operating characteristic curve analyses were conducted to evaluate the resistance prediction models for (A) amoxicillin and (B) rifamycin.

# Supplementary Tables

## Supplementary Table 1. Demographic and clinical characteristics of study participants

|  | **All pariticipants**  **n = 468** | ***H. pylori* culture-positive participants**  **n = 165** | **Participants involved in final analysis**  **n = 153** |
| --- | --- | --- | --- |
| Age (year) | 55.01±8.44 | 53.39±8.52 | 53.65±8.71 |
| Sex (%) |  |  |  |
| Male | 218（46.58） | 73（44.25） | 67（43.79） |
| Female | 250（53.42） | 92（52.15） | 86（56.21） |
| History of smoking (%) |  |  |  |
| Yes | 140（29.91） | 46（27.88） | 42（27.45） |
| No | 328（70.09） | 119（72.12） | 111（72.55） |
| History of drinking alcohol (%) |  |  |  |
| Yes | 139（29.70） | 49（29.70） | 46（30.07） |
| No | 329（70.30） | 116（70.30） | 107（69.93） |
| Gastric mucosal diagnoses (%) |  |  |  |
| Superficial gastritis | 324（69.23） | 120（72.73） | 113（73.86） |
| Chronic atrophy gastritis | 96（20.51） | 64（21.12） | 27（17.65） |
| Intestinal metaplasia | 37（7.91） | 27（8.91） | 10（6.54） |
| Dysplasia | 5（1.07） | 3（0.99） | 2（1.30） |
| Missing | 6（1.28） | 5（1.65） | 1（0.65） |

## Supplementary Table 2. Candidate literature-reported antibiotic resistance genes and mutations

| **Antibiotics^a^** | **Gene names** | **Candidate mutations ^b^** |
| --- | --- | --- |
| AC | *pbp1* | V45I, N107R, A201V, V250I, T337_S338insN, S338R, F366L, V374L, Y401_S402insY, S402G, N404S, S405N, S414R, L423F, I450V, S455N, K464_D465insE/K/D, V469M/A, A474T, N504D, D535N, S543H/R/T, T556S, T558S, N562D/H/Y, T593A/G/K/P/S, G595del/A/S, A599T/P/V, L610STOP, Y637STOP |
|  | *pbp2* | A296V, V312M, V313A, G353R, S494H, A541M, E572G, I259T |
|  | *pbp3* | D2N, A50S, F233L, V374L, F490Y, A499V, E536K, A54IT |
| CH | *23S rRNA* | A1410G, C1707T, G1939A, T1942C, G2111A, A2115G, A2116G, A2142C/G, A2143G, A2144G/T, A2146C/G/U, A2147C/G, C2147G, A2167G, G2172T, T2182C, G2224A, C2245T, C2248T, G2287A |
| LE | *gyrA* | D34Y, H57Y, S63P, V65I, V77A, S83A, G85C, D86N, N87A/K/I/Y/T, A88N/P/V, D91G/N/A/H/Y, A92T, A97V, D99V, R103H, A129T, R130K, R140K, D155N, D161N, V172I, P188S, D192N, A199V/I |
|  | *gyrB* | D435N, V437L, F438S, S429T, E463K, S479G, D481E, D484K, R484K, R579C |
| MZ | *frxA* | R3T, V7I, Q5STOP, A15V, A16T, A32V, L39STOP, P41L, S43A, V44G, I44F, Y60F, Y62D, M66I, A70V, A85V, R86STOP, K105STOP, R106frameshift, I117M, M126F, I144V, M149STOP, A192STOP, E199STOP |
|  | *rdxA* | A1G, Q5STOP, L13frameshift, R16C/H, C18frameshift, M21V, E27V, T31E, T38frameshift, G47A, C49T, Q50STOP, A50STOP, D59N/S, D59STOP, G60A, T62frameshift, K64N, A67V, A68E, A70V, N73STOP, A80T, R90K, A96frameshift, H97T/Y, P106S, S108A, G112frameshift, A118S/T, A119frameshift, C148Y, T162frameshift, R176C, T184G, G189C/S, S196N, L210V, C273T |
| TC | *16S rRNA* | AGA926_928TTC/GGC, AG926_927GT, A926C/G/T, GA927_928TC, G927T, A928C, A939C, A965G/U, G966U, A967C |
| RI | *rpoB* | V148I, V149F/W/D/E/Q/K, Q524P, L525P/I, Q527R/K, D530N/E/G/F/I/V/Y/L, V538I, H540N/Y, S545L, I586L/N, A603T, R701H, I837V, K2068R, Q2079K, A2414V, A2414T, T2537A, F2538L, V2592L, I2619V |

1. AC, amoxicillin, CH, clarithromycin; LE, levofloxacin; MZ, metronidazole; RI, rifamycin; TC, tetracycline.
2. Mutations are reported following standard recommendations in molecular diagnostics from the Human Genome Variation Society. For genes coding for proteins, mutations in codons were described; for genes coding for RNA, mutations in the bases of the coding genes were described. Examples: V45I (substitution with missense change); Y401_S402insY (in-frame insertions); L13frameshift (frame shift mutation); Q5STOP (nonsense mutation or premature stop codon). Nucleotide residues in DNA sequences are represented by heterocyclic bases as follows: A for adenine, C for cytosine, G for guanine, and T for thymine. Single-letter abbreviations for amino acid residues in protein sequences include: A for alanine, C for cysteine, D for aspartic acid, E for glutamic acid, F for phenylalanine, G for glycine, H for histidine, I for isoleucine, K for lysine, L for leucine, M for methionine, N for asparagine, P for proline, Q for glutamine, R for arginine, S for serine, T for threonine, V for valine, W for tryptophan, and Y for tyrosine.
3. For genes coding RNA, the base positions of the mutations were described. Nucleotide residues in DNA sequences are represented by heterocyclic bases as A for adenine, C for cytosine, G for guanine, and T for thymine.

## Supplementary Table 3. Significant antibiotic resistance associated SNPs and k-mers by GWAS

| **Antibiotics** | **Variants** | **Position^a^** | ***P* ^b^** | **Antibiotic resistance genes^c^** | **k-mers sequences** |
| --- | --- | --- | --- | --- | --- |
| CH | kmer-1 | 447365-447424 | 7.03E-15 | *HP_r01* | CTAAGTTGTAGTAAAGGTCCACGGGGTCTCTCCGTCTTGCCGCGGGTAGGAGGAATTTTC |
|  |  | 1474777-1474718 |  | *HP_r06* |  |
|  | kmer-2 | 447425-447396 | 2.12E-14 | *HP_r01* | GAGACCCCGTGGACCTTTACTACAACTTAGC |
|  |  | 1474717-1474746 |  | *HP_r06* |  |
|  | kmer-3 | 447402-447365 | 2.93E-12 | *HP_r01* | CTCCTACCCGCGGCAAGACGGAAAGACCCCGTGGACCTTTA |
|  |  | 1474740 -1474777 |  | *HP_r06* |  |
|  | kmer-4 | 447413-447373 | 2.93E-12 | *HP_r01* | GAAAATTCCTCCTACCCGCGGCAAGACGGAAAGACCCC |
|  |  | 1474729-1474769 |  | *HP_r06* |  |
|  | kmer-5 | 447424-447384 | 6.45E-12 | *HP_r01* | GGCAAGACGGAAAGACCCCGTGGACCTTTACTACAACTTAG |
|  |  | 1474718-1474758 |  | *HP_r06* |  |
|  | kmer-6 | 447425-447395 | 7.06E-12 | *HP_r01* | AAGACCCCGTGGACCTTTACTACAACTTAGC |
|  |  | 1474717-1474747 |  | *HP_r06* |  |
|  | kmer-7 | 1234832-1234865 | 5.44E-07 | *HP1168* | AATGTTTATAAGCCTTTGGGCAATCTTAGCTCTT |
|  | kmer-8 | 1234854-1234824 | 5.88E-07 | *HP1168* | AGCCTTATAAGAGCTAAGATTGCCCAAAGGC |
|  | kmer-9 | 1234825-1234856 | 5.88E-07 | *HP1168* | AAGCCTTTGGGCAATCTTAGCTCTTATAAGGC |
|  | kmer-10 | 1234827-1234861 | 1.10E-06 | *HP1168* | TTTATAAGCCTTTGGGCAATCTTAGCTCTTATAAG |
|  | kmer-11 | 607304-607268 | 1.86E-06 | *HP0576* | AAAAAAGATAACCAACAGCACAATAATAATCGTCCCT |
|  | kmer-12 | 1234866-1234836 | 5.54E-06 | *HP1168* | GCTAAGATTGCCCAAAGGCTTATAAACATTA |
|  | kmer-13 | 844874-844843 | 6.12E-06 | *HP0788* | AACACCGTTTATGCAAACACCCTTAACTCTTT |
|  | kmer-14 | 1101775-1101804 | 6.92E-06 | *HP1041* | TTGGTGCGCTGTTTGATGTTGTTTGCAAGCAC |
|  | kmer-15 | 1387806-1387774 | 6.99E-06 | *HP1328* | AGAAAAATTAAAACTATTAGGGCTAGAAAACTC |
|  | kmer-16 | 70292-70261 | 8.62E-06 | *HP0066* | ATAATCAATCCGCAGAAGATTTGAAACGCTAT |
|  | kmer-17 | 1418484-1418515 | 8.84E-06 | *HP1356* | GCTAAAGATGGTGGCGTGGTTTGCACGAGCCG |
|  | kmer-18 | 1249122-1249092 | 8.91E-06 | *HP1180* | CTTTTATTGCTAGAAAACACCCAAGCAATAA |
|  | kmer-19 | 1304113-1304081 | 9.97E-06 | *HP1229* | GCGCATTTGTGCGAAATCTATACGGATGTGGAT |
| LE | kmer-20 | 1493596-1493566 | 3.42E-07 | *HP1422* | ACATGCTCGCCCAGATAGCTTTCATCTAATA |
|  | kmer-21 | 412202-412154 | 7.63E-07 | *HP0401* | GCGAAACTCATCGCAATCCTATGATCGTTAAAGCTTTGGATAAGGGGGG |
|  | kmer-22 | 1493595-1493562 | 1.97E-06 | *HP1422* | AAAAACATGCTCGCCCAGATAGCTTTCATCTAAT |
|  | kmer-23 | 700104-700134 | 2.65E-06 | *HP0654* | AAGATCCCACCTTACCATTACAGATTTTACG |
|  | kmer-24 | 1408165-1408194 | 2.71E-06 | *HP1348* | CTAGATCAAAACCGCCCTTTAGTGATTTTCC |
|  | kmer-25 | 700105-700136 | 3.10E-06 | *HP0654* | AGAAGATCCCACCTTACCATTACAGATTTTAC |
|  | kmer-26 | 218837-218866 | 3.14E-06 | *HP0211* | TTTTGAAAAAGCTTGCGATTTGAATAATGGTGG |
|  | kmer-27 | 915585-915547 | 3.24E-06 | *HP0863* | TCTAAAGGCTGTTTATTTTCTTTGACTTTATGCCTTTCT |
|  | kmer-28 | 758047-758077 | 3.25E-06 | *HP0705* | GCATTTGGATAAAGTGATTTATTTAGACCAA |
|  | kmer-29 | 1493591-1493560 | 3.57E-06 | *HP1422* | TTAAAAACATGCTCGCCCAGATAGCTTTCATC |
|  | kmer-30 | 700148-700118 | 4.96E-06 | *HP0654* | TGGTAAGGTGGGATCTTCTCGGTGGTTAGAA |
|  | kmer-31 | 758046-758076 | 5.99E-06 | *HP0705* | CATTTGGATAAAGTGATTTATTTAGACCAAG |
|  | kmer-32 | 218837-218867 | 6.27E-06 | *HP0211* | TATTTTGAAAAAGCTTGCGATTTGAATAATGG |
|  | kmer-33 | 1493596-1493567 | 6.50E-06 | *HP1422* | CATGCTCGCCCAGATAGCTTTCATCTAATAAT |
|  | kmer-34 | 1159452-1159416 | 6.52E-06 | *HP1098* | TATTCAAATCGCAAGCTTTTTCAAAATACTTCCTAGCTT |
|  | kmer-35 | 700101-700133 | 7.68E-06 | *HP0654* | AGATCCCACCTTACCATTACAGATTTTACGCCT |
|  | kmer-36 | 758048-758079 | 8.45E-06 | *HP0705* | GAGCATTTGGATAAAGTGATTTATTTAGACCA |
|  | kmer-37 | 638689-638721 | 8.60E-06 | *HP0601* | ATTCGCACTCTCTTCAGCAAAATCCACATCCCT |
|  | kmer-38 | 638694-638726 | 8.60E-06 | *HP0601* | TTGAAATTCGCACTCTCTTCAGCAAAATCCACA |
|  | SNP  (1408195 A>G) | 1408195 | 7.89E-06 | *HP1348* |  |
|  | SNP  (1493579 G>A) | 1493579 | 4.65E-06 | *HP1422* |  |
| MZ | kmer-39 | 1388611-1388579 | 1.00E-07 | *HP1329* | AACCAAAGCCCTAAAATCGTGCAAGAGCAGGTT |
|  | kmer-40 | 1444678-1444709 | 8.03E-07 | *HP1379* | AAAGTCAATTCGCCCGTCATTGCCACTTCGCT |
|  | kmer-41 | 1444681-1444712 | 8.07E-07 | *HP1379* | CTCAAAGTCAATTCGCCCGTCATTGCCACTTC |
|  | kmer-42 | 266446-266485 | 1.11E-06 | *HP0257* | GCAAATCGTCTTTCAATTCGGCTTTTTTAGATTCAAAAAT |
|  | kmer-43 | 120446-120414 | 1.32E-06 | *HP0112* | TTCTTTGGGCGAAGAAATCGCCATTTTTAACCC |
|  | kmer-44 | 120443-120406 | 1.35E-06 | *HP0112* | GGGTATCGTTCTTTGGGCGAAGAAATCGCCATTTTTAA |
|  | kmer-45 | 1321409-1321375 | 1.53E-06 | *HP1246* | CAAAGTCAATTCGCCCGTCATTGCCACTTCG |
|  | kmer-46 | 1444680-1444710 | 1.53E-06 | *HP1379* | TCTAAGATCATTGACGGCTCTGCTTTGAAATACGC |
|  | kmer-47 | 1321394-1321360 | 2.28E-06 | *HP1246* | TACAATCGTTCAAGCTCTAAGATCATTGACGGCTC |
|  | kmer-48 | 1321389-1321357 | 2.31E-06 | *HP1246* | CGATACAATCGTTCAAGCTCTAAGATCATTGAC |
|  | kmer-49 | 431591-431620 | 2.35E-06 | *HP0417* | CGCGCTCGCATAATTCAATAAAGCGTCCAAC |
|  | kmer-50 | 1444669-1444707 | 2.50E-06 | *HP1379* | AGGGTATCGTTCTTTGGGCGAAGAAATCGCC |
|  | kmer-51 | 1321404-1321365 | 3.15E-06 | *HP1246* | AGTCAATTCGCCCGTCATTGCCACTTCGCTTCTTGTAGC |
|  | kmer-52 | 120450-120417 | 3.18E-06 | *HP0112* | TCGTTCAAGCTCTAAGATCATTGACGGCTCTGCTTTGAAA |
|  | kmer-53 | 334075-334045 | 3.25E-06 | *HP0317* | TTTGGGCGAAGAAATCGCCATTTTTAACCCCAAA |
|  | kmer-54 | 1320023-1319993 | 3.25E-06 | *HP1243* | GAAACTAAAAGCGAGCCTAAAGCTAATGAAA |
|  | kmer-55 | 1444668-1444698 | 3.44E-06 | *HP1379* | GCCCGTCATTGCCACTTCGCTTCTTGTAGCC |
|  | kmer-56 | 808629-808595 | 4.82E-06 | *HP0752* | AGCGATAGCAAGGATATGGGGGGCAGAGAAATCCA |
|  | kmer-57 | 1444665-1444697 | 4.90E-06 | *HP1379* | CCCGTCATTGCCACTTCGCTTCTTGTAGCCCTA |
|  | kmer-58 | 266443-266475 | 5.23E-06 | *HP0257* | TTTCAATTCGGCTTTTTTAGATTCAAAAATCGC |
|  | kmer-59 | 1388604-1388555 | 5.49E-06 | *HP1329* | GTGGTCGTGCAAATCACTTACCCCAATCAAAGCCCTAAAATCGTGCAAGA |
|  | kmer-60 | 808624-808594 | 6.09E-06 | *HP0752* | GAGCGATAGCAAGGATATGGGGGGCAGAGAA |
|  | kmer-61 | 846446-846416 | 6.51E-06 | *HP0790* | ATTTTTTTTGCACCATTCCCCCAAAAGAAAA |
|  | kmer-62 | 202265-202231 | 7.26E-06 | *HP0195* | TTAGGCAAGCATAACATAAGGGTGAATGCCCTATC |
|  | kmer-63 | 1444713-1444683 | 8.22E-06 | *HP1379* | AGTGGCAATGACGGGCGAATTGACTTTGAGC |
|  | kmer-64 | 1011905-1011942 | 8.47E-06 | *HP0951* | AAATAGATACTATCCAAAGAATGCACGCCCTCTAAATG |
|  | kmer-65 | 783368-783333 | 8.65E-06 | *HP0728* | TATCCTTTTCAATGATGAATTTTTGAGCGATTTCTA |
|  | kmer-66 | 1321386-1321350 | 8.94E-06 | *HP1246* | ATTGATGCGATACAATCGTTCAAGCTCTAAGATCATT |
|  | SNP  (1321380 A>G) | 1321380 | 1.04E-06 | *HP1246* |  |
|  | SNP  (1388581 T>C) | 1388581 | 5.49E-06 | *HP1329* |  |
| RI | kmer-67 | 271436-271472 | 6.06E-07 | *HP0262* | GCAAAACGCTATATTCTCCAAAATGCACCAATAAAGCCC |
|  | kmer-68 | 271465-271435 | 1.10E-06 | *HP0262* | CGGGCTTTATTGGTGCATTTTGGAGAATATA |
|  | kmer-69 | 1083015-1083045 | 2.62E-06 | *HP1020* | GGTTTGAGAAAATCGTTTCGATTCCCCAGCA |
|  | kmer-70 | 262564-262534 | 2.83E-06 | *HP0252* | TCAGCTTATCTATAAGAAAGGGCTAGACAGA |
|  | kmer-71 | 1358710-1358740 | 3.73E-06 | *HP1282* | CATTCCCTATCCCCTAGCTCTTTATGAAAAA |
|  | kmer-72 | 780714-780743 | 5.30E-06 | *HP0725* | TTGGTCAATAGCTCTTTAAACGATTTAAAAA |
|  | kmer-73 | 233369-233339 | 5.46E-06 | *HP0224* | AGTGTTGAGCCGTATTGGTAAGGCGCATTTA |
|  | kmer-74 | 271430-271464 | 6.98E-06 | *HP0262* | ATATTCTCCAAAATGCACCAATAAAGCCCGTTTGA |
|  | kmer-75 | 775482-775510  779857-779839 | 9.29E-06 | *HP0722*  *HP0725* | CCGATTGCCATAATAACTCACATTGTTTTGA |
| TC | kmer-76 | 1493891-1493859 | 8.95E-07 | *HP1422* | AGCGGCTAACTTTTCATGCAAGGCTTTAGCGAC |
|  | kmer-77 | 1288547-1288580 | 3.34E-06 | *HP1213* | GATTAGTTTCAATAAGGGTAAAATCGCTTTAGAT |
|  | kmer-78 | 990369-990334 | 4.48E-06 | *HP0927* | TATCGCGCAAAACAGACTCAAAACGAACGCAGTTTT |
|  | kmer-79 | 1493867-1493838 | 5.58E-06 | *HP1422* | CTCACTATCCACCACCCCTAAAGCGGCTAAC |
|  | kmer-80 | 990379-990340 | 5.68E-06 | *HP0927* | GCAAAACAGACTCAAAACGAACGCAGTTTTAGCGACTTAT |
|  | kmer-81 | 64179-64149 | 9.66E-06 | *HP0060* | AAGAAGATAAAATCAAGTTCAAGCAAGTGCT |
|  | SNP (125771 A>G) | 125771 | 8.69E-06 | *HP0116* |  |

1. Corresponding position on reference strain, *H.pylori 26695*.
2. The *P*-value of GWAS analysis, adjusted for population structure.
3. Corresponding gene locus on *H.pylori 26695*.

CH, clarithromycin, LE, levofloxacin, MZ, metronidazole, RI, rifamycin, TC, tetracycline.

## Supplementary Table 4. Associations between *HefA* mutations and antibiotic resistance phenotypes

| **Antibiotics** | **Mutation sites^a^** | **Genotype** | **Resistance phenotype (%)** | | ***P*** | **MIC  median (interquartile)** | ***P^d^*** |
| --- | --- | --- | --- | --- | --- | --- | --- |
|  |  |  | **Sensitive** | **Resistant** |  |  |  |
| AC | N2K/R | W | 3(100.00) | 0 (0.00) | 1.00^b^ | 0.015 (0.015, 0.015) | 0.18 |
|  |  | M | 142 (95.95) | 6 (4.05) |  | 0.015 (0.015, 0.032) |  |
|  | A8V | W | 35 (97.22) | 1 (2.78) | 1.00^b^ | 0.015 (0.015, 0.032) | 0.89 |
|  |  | M | 110 (95.65) | 5 (4.35) |  | 0.015 (0.015, 0.032) |  |
|  | A15T/V | W | 139(95.86) | 6 (4.14) | 1.00^b^ | 0.015 (0.015, 0.032) | 0.74 |
|  |  | M | 6(100.00) | 0 (0.00) |  | 0.015 (0.015, 0.032) |  |
|  | A16T/V | W | 90 (93.75) | 6 (6.25) | 0.09^b^ | 0.015 (0.015, 0.032) | 0.07 |
|  |  | M | 55 (100.00) | 0 (0.00) |  | 0.015 (0.015, 0.016) |  |
|  | T18A | W | 58 (95.08) | 3 (4.92) | 0.69^b^ | 0.015 (0.015, 0.032) | 0.31 |
|  |  | M | 87 (96.67) | 3 (3.33) |  | 0.015 (0.015, 0.023) |  |
|  | S61Q | W | 117 (95.90) | 5 (4.10) | 1.00^b^ | 0.015 (0.015, 0.032) | 0.8 |
|  |  | M | 28 (96.55) | 1 (3.45) |  | 0.015 (0.015, 0.040) |  |
|  | R229K | W | 9 (0.00) | 0 (0.00) | 1.00^b^ | 0.015 (0.015, 0.040) | 0.8 |
|  |  | M | 138 (95.83) | 6 (4.17) |  | 0.015 (0.015, 0.032) |  |
|  | S272N | W | 132 (97.06) | 4 (2.94) | 0.13^b^ | 0.015 (0.015, 0.023) | 0.16 |
|  |  | M | 15 (88.24) | 5 (11.76) |  | 0.015 (0.015, 0.064) |  |
|  | A283V | W | 9 (100.00) | 0 (0.00) | 1.00^b^ | 0.015 (0.015, 0.032) | 0.76 |
|  |  | M | 138 (95.83) | 6 (4.17) |  | 0.015 (0.015, 0.032) |  |
| CH | N2K/R | W | 1 (33.33) | 2 (66.67) | 1.00^b^ | 0.500 (0.313, 128.750) | 0.87 |
|  |  | M | 54 (36.49) | 94 (63.51) |  | 1.250 (0.125, 28.000) |  |
|  | A8V | W | 13 (36.11) | 23 (63.89) | 0.96^c^ | 0.750 (0.125, 30.000) | 0.93 |
|  |  | M | 42 (36.52) | 73 (63.48) |  | 1.500 (0.125, 32.000) |  |
|  | A15T/V | W | 53 (36.55) | 92 (62.45) | 1.00^b^ | 1.000 (0.125, 28.000) | 0.55 |
|  |  | M | 2 (33.33) | 4 (66.67) |  | 16.000 (0.174, 48.000) |  |
|  | A16T/V | W | 36 (37.50) | 60 (62.50) | 0.72^c^ | 0.625 (0.125, 24.000) | 0.24 |
|  |  | M | 19 (34.55) | 36 (65.45) |  | 2.000 (0.190, 48.000) |  |
|  | T18A | W | 24 (39.34) | 37 (60.66) | 0.54^c^ | 1.500 (0.125, 40.000) | 0.88 |
|  |  | M | 31 (34.44) | 59 (65.56) |  | 1.000 (0.190, 24.000) |  |
|  | S61Q | W | 45(36.89) | 77 (63.11) | 0.81^c^ | 0.875 (0.125, 24.000) | 0.53 |
|  |  | M | 10 (34.48) | 19 (65.52) |  | 2.000 (0.190, 48.000) |  |
|  | R229K | W | 3 (33.33) | 6 (66.67) | 1.00^b^ | 16.000 (0.220, 160.500) | 0.27 |
|  |  | M | 53 (36.81) | 91 (63.19) |  | 1.000 (0.125, 26.000) |  |
|  | S272N | W | 53 (38.97) | 83 (61.03) | 0.09^c^ | 0.625 (0.125, 24.000) | 0.14 |
|  |  | M | 3 (17.65) | 14 (82.35) |  | 16.000 (0.440, 80.000) |  |
|  | A283V | W | 4 (44.44) | 5 (55.56) | 0.73^c^ | 0.380 (0.056, 14.000) | 0.12 |
|  |  | M | 52 (36.11) | 92 (63.89) |  | 1.500 (0.125, 32.000) |  |
| LE | N2K/R | W | 0 (0.00) | 3 (100.00) | 1.00^b^ | 33.000 (18.500, 33.000) | 0.2 |
|  |  | M | 30 (20.27) | 118 (79.73) |  | 4.000 (1.500, 33.000) |  |
|  | A8V | W | 9 (25.00) | 27 (75.00) | 0.38^c^ | 3.500 (1.125, 33.000) | 0.54 |
|  |  | M | 21 (18.26) | 94 (81.74) |  | 4.000 (1.500, 33.000) |  |
|  | A15T/V | W | 29 (20.00) | 116 (80.00) | 1.00^b^ | 4.000 (1.500, 33.000) | 0.37 |
|  |  | M | 1 (16.67) | 5 (83.33) |  | 33.000 (1.625, 33.000) |  |
|  | A16T/V | W | 21 (21.88) | 75 (78.12) | 0.41^c^ | 4.000 (1.500, 33.000) | 0.78 |
|  |  | M | 9 (16.36) | 46 (83.64) |  | 3.000 (1.500, 33.000) |  |
|  | T18A | W | 12 (19.67) | 49 (80.33) | 0.96 ^c^ | 4.000 (1.500, 33.000) | 0.84 |
|  |  | M | 18 (20.00) | 72 (80.00) |  | 3.500 (1.500, 33.00) |  |
|  | S61Q | W | 25 (20.49) | 97 (79.51) | 0.69 ^c^ | 4.000 (1.500, 33.000) | 0.29 |
|  |  | M | 5 (17.24) | 24 (82.76) |  | 33.000 (1.500, 33.000) |  |
|  | R229K | W | 1 (11.11) | 8 (88.89) | 1.00^b^ | 4.000 (1.500, 33.000) | 0.85 |
|  |  | M | 29 (20.14) | 115 (79.86) |  | 4.000 (1.500, 33.000) |  |
|  | S272N | W | 26 (19.12) | 110 (80.88) | 0.75^b^ | 4.000 (1.500, 33.000) | 0.5 |
|  |  | M | 4 (23.53) | 13 (76.47) |  | 33.000 (1.250, 33.000) |  |
|  | A283V | W | 3 (33.33) | 6 (66.67) | 0.38^b^ | 3.000 (1.000, 33.000) | 0.57 |
|  |  | M | 27 (18.75) | 117 (81.25) |  | 4.000 (1.500, 33.000) |  |
| MZ | N2K/R | W | 0 (0.00) | 3 (100.00) | 1.00^b^ | 257.000 (160.500, 257.000) | 0.51 |
|  |  | M | 31 (20.95) | 117 (79.05) |  | 257.000 (32.000,257.000) |  |
|  | A8V | W | 8 (22.22) | 28 (77.78) | 0.77^c^ | 224.000 (32.000, 257.000) | 0.59 |
|  |  | M | 23 (20.00) | 92 (80.00) |  | 257.000 (32.000, 257.000) |  |
|  | A15T/V | W | 28 (19.31) | 117 (80.69) | 0.10^b^ | 257.000 (32.000, 257.000) | 0.44 |
|  |  | M | 3 (50.00) | 3 (50.00) |  | 130.500 (0.875, 257.000) |  |
|  | A16T/V | W | 21 (21.88) | 75 (78.12) | 0.59^c^ | 257.000 (36.000, 257.000) | 0.53 |
|  |  | M | 10 (18.18) | 45 (81.82) |  | 256.000 (32.000, 257.000) |  |
|  | T18A | W | 13 (21.31) | 48 (78.69) | 0.84^c^ | 257.000 (40.000, 257.000) | 0.91 |
|  |  | M | 18 (20.00) | 72 (80.00) |  | 257.000 (32.000, 257.000) |  |
|  | S61Q | W | 26 (21.31) | 96 (78.12) | 0.63^c^ | 257.000 (32.000, 257.000) | 0.36 |
|  |  | M | 5 (17.24) | 24 (82.76) |  | 257.000 (48.000, 257.000) |  |
|  | R229K | W | 5 (55.56) | 4 (44.44) | **0.017**^b^ | 4.000 (1.375, 160.500) | **0.012** |
|  |  | M | 26 (18.06) | 118 (81.94) |  | 257.000 (44.000, 257.000) |  |
|  | S272N | W | 26 (19.12) | 110 (80.88) | 0.34^c^ | 257.000 (32.000, 257.000) | 0.21 |
|  |  | M | 5 (29.41) | 12 (70.39) |  | 96.000 (4.375, 257.000) |  |
|  | A283V | W | 1 (11.11) | 8 (89.89) | 0.69^c^ | 257.000 (40.000, 257.000) | 0.95 |
|  |  | M | 30 (20.83) | 114 (79.17) |  | 257.000 (32.000, 257.000) |  |
| RI | N2K/R | W | 1 (33.33) | 2 (66.67) | 0.57^c^ | 1.500 (0.940, 4.750) | 0.62 |
|  |  | M | 89 (60.13) | 59 (39.87) |  | 1.000 (0.500, 2.000) |  |
|  | A8V | W | 22 (61.11) | 14 (38.89) | 0.83^c^ | 1.000 (0.500, 2.000) | 0.61 |
|  |  | M | 68 (59.13) | 47 (40.87) |  | 1.000 (0.500, 2.000) |  |
|  | A15T/V | W | 86 (59.31) | 59 (40.69) | 1.00^b^ | 1.000 (0.500, 2.000) | 0.66 |
|  |  | M | 4 (66.67) | 2 (33.33) |  | 1.000 (0.658, 2.500) |  |
|  | A16T/V | W | 57 (59.38) | 39 (40.63) | 0.94^c^ | 1.000 (0.500, 2.000) | 0.83 |
|  |  | M | 33 (60.00) | 22 (40.00) |  | 1.000 (0.500, 2.000) |  |
|  | T18A | W | 36 (59.02) | 25 (40.98) | 0.9^c^ | 1.000 (0.500, 2.000) | 0.47 |
|  |  | M | 54 (60.00) | 36 (40.00) |  | 1.000 (0.500, 2.000) |  |
|  | S61Q | W | 76 (62.30) | 46 (37.70) | 0.17^c^ | 1.000 (0.500, 2.000) | 0.15 |
|  |  | M | 14 (48.28) | 15 (51.72) |  | 1.500 (0.500, 2.000) |  |
|  | R229K | W | 84 (58.33) | 60 (41.67) | 0.74 ^c^ | 1.000 (0.625, 1.750) | 0.92 |
|  |  | M | 6 (66.67) | 3 (33.33) |  | 1.000 (0.500, 2.000) |  |
|  | S272N | W | 79 (58.09) | 57 (41.91) | 0.60^c^ | 1.000 (0.500, 2.000) | 0.61 |
|  |  | M | 11 (64.71) | 6 (35.29) |  | 1.000 (0.440, 1.750) |  |
|  | A283V | W | 5 (55.56) | 4 (44.44) | 1.00^b^ | 1.000 (0.500, 1.750) | 0.97 |
|  |  | M | 85 (59.03) | 59 (40.97) |  | 1.000 (0.500, 2.000) |  |
| TC | N2K/R | W | 3 (100.00) | 0 (0.00) | 1.00^b^ | 1.000 (0.875, 1.000) | 0.16 |
|  |  | M | 124 (83.78) | 24 (16.22) |  | 0.500 (0.250, 1.000) |  |
|  | A8V | W | 28 (77.78) | 8 (22.22) | 0.23^c^ | 0.625 (0.380, 1.000) | 0.37 |
|  |  | M | 99 (86.09) | 16 (13.91) |  | 0.500 (0.250, 1.000) |  |
|  | A15T/V | W | 123 (84.83) | 22 (15.17) | 0.24^c^ | 0.500 (0.250, 1.000) | 0.15 |
|  |  | M | 4 (66.67) | 2 (33.33) |  | 1.000 (0.438, 1.875) |  |
|  | A16T/V | W | 77 (80.21） | 19 (19.79) | 0.084^c^ | 0.625 (0.283, 1.000) | 0.25 |
|  |  | M | 50 (90.91) | 5 (9.09) |  | 0.500 (0.250, 0.750) |  |
|  | T18A | W | 48 (78.69) | 13 (21.31) | 0.13^c^ | 0.750 (0.250, 1.000) | 0.45 |
|  |  | M | 79 (87.78) | 11 (12.22) |  | 0.500 (0.348, 0.750) |  |
|  | S61Q | W | 102 (83.61) | 20 (16.39) | 1.00^b^ | 0.500 (0.250, 1.000) | 0.61 |
|  |  | M | 25 (86.21) | 4 (13.79) |  | 0.500 (0.380, 1.000) |  |
|  | R229K | W | 8 (88.89) | 1 (11.11) | 1.00^b^ | 0.500 (0.380, 0.750) | 0.73 |
|  |  | M | 121 (84.03) | 23 (16.98) |  | 0.500 (0.250, 1.000) |  |
|  | S272N | W | 116 (85.29) | 20 (14.71) | 0.31^c^ | 0.500 (0.250, 1.000) | 0.88 |
|  |  | M | 13 (76.47) | 4 (23.53) |  | 0.500 (0.315, 1.875 |  |
|  | A283V | W | 9 (100.00) | 0 (0.00) | 0.36^c^ | 0.250 (0.250, 0.500) | **0.044** |
|  |  | M | 120 (83.33) | 24 (16.67) |  | 0.500 (0.348, 1.000) |  |

1. Mutations are reported following standard recommendations in molecular diagnostics from the Human Genome Variation Society. For genes coding proteins, mutations in codons were described. A substitution with missense change (e.g. K229R) is described by the residue in the wild type strain, the sequence position and the residue in the mutant type strain. In-frame insertions (e.g. K464_D465insE/K/D) are described using ‘ins’ after an indication of the residue flanking the insertion site, separated by ‘_’ (underscore) and followed by a description of the residue inserted. Single-letter abbreviations for amino acid residues in protein sequences include A for alanine, C for cysteine, D for aspartic acid, E for glutamic acid, F for phenylalanine, G for glycine, H for histidine, I for isoleucine, K for lysine, L for leucine, M for methionine, N for asparagine, P for proline, Q for glutamine, R for arginine, S for serine, T for threonine, V for valine, W for tryptophan, and Y for tyrosine.
2. Fisher’s exact test for the comparison of resistance rate between wild and mutant type strains.
3. Chi-square test for the comparison of resistance rate between wild and mutant type strains
4. Man-Whitney test for the comparison of MIC between wild and mutant type strains.

AC, amoxicillin, CH, clarithromycin, LE, levofloxacin, MZ, metronidazole, RI, rifamycin , TC, tetracycline, W, wild type, M, Mutant type, MIC, minimum inhibitory concentration

## Supplementary Table 5. Associations between literature-reported mutations and antibiotic resistance phenotypes

| **Antibiotics** | **Resistance genes** | **Mutation sites** | **Genotype** | **Resistance phenotype (%)** | | ***P*** | **MIC  median (interquartile)** | ***P ^e^*** |
| --- | --- | --- | --- | --- | --- | --- | --- | --- |
|  |  |  |  | **Sensitive** | **Resistant** |  |  |  |
| AC | *pbp1* | V45N^a^ | W | 133 (98.68) | 6 (4.32) | 1.00^d^ | 0.015 (0.015, 0.032) | 0.07 |
|  |  |  | M | 12 (100.00) | 0 (0.00) |  | 0.015 (0.015, 0.015) |  |
|  |  | V250I^a^ | W | 135 (96.43) | 5 (3.57) | 0.37^d^ | 0.015 (0.015, 0.023) | 0.36 |
|  |  |  | M | 10 (90.91) | 1 (9.09) |  | 0.023 (0.015, 0.047) |  |
|  |  | K464_D465insE/K/D^a^ | W | 142 (97.93) | 3 (2.07) | **0.001**^d^ | 0.015 (0.015, 0.023) | **<0.001** |
|  |  |  | M | 4 (57.14) | 3 (42.86) |  | 0.125 (0.047, 0.250) |  |
|  |  | A474T/V^a^ | W | 139 (96.53) | 5 (3.47) | 0.28^d^ | 0.015 (0.015, 0.032) | 0.98 |
|  |  |  | M | 7 (87.5) | 1 (12.5) |  | 0.015 (0.015, 0.030) |  |
|  |  | N504D^a^ | W | 14 (100.00) | 0 (0.00) | 1.00^d^ | 0.015 (0.015, 0.036) | 0.72 |
|  |  |  | M | 132 (95.65) | 6 (4.35) |  | 0.015 (0.015, 0.023) |  |
|  |  | T593A/G/K/P/S^a^ | W | 124 (98.41) | 2 (1.59) | **0.009**^d^ | 0.015 (0.015, 0.016) | **<0.001** |
|  |  |  | M | 23 (85.19) | 4 (14.81) |  | 0.032 (0.016, 0.072) |  |
|  |  | G595S^a^ | W | 92 (97.87) | 2 (2.13) | 0.21^d^ | 0.015 (0.015, 0.016) | **<0.001** |
|  |  |  | M | 55 (93.22) | 4 (6.78) |  | 0.016 (0.015, 0.032) |  |
| CH | *23S rRNA* | A2143G^b^ | W | 54 (59.34) | 37 (40.66) | **<0.001**^d^ | 0.190 (0.125, 0.500) | **<0.001** |
|  |  |  | M | 2 (3.64) | 53 (96.36) |  | 24.000 (16.000, 64.000) |  |
|  |  | T2182C^b^ | W | 5 (38.46) | 8 (61.54) | 1.00^d^ | 3.000 (0.094, 24.000) | 0.86 |
|  |  |  | M | 51 (36.69) | 88 (63.31) |  | 0.500 (0.125, 24.000) |  |
|  |  | A2223G^b^ | W | 55 (38.19) | 89 (61.81) | 0.26 ^d^ | 0.500 (0.125, 24.000) | 0.15 |
|  |  |  | M | 1 (12.5) | 7 (87.5) |  | 12.000 (2.563, 196.756) |  |
|  |  | C2922T^b^ | W | 53 (37.32) | 89 (62.68) | 1.00^d^ | 0.500 (0.125, 24.000) | 0.35 |
|  |  |  | M | 3 (42.86) | 4 (57.14) |  | 0.380 (0.094, 12.000) |  |
| LE | *gyrA* | N87K/R^a^ | W | 28 (22.58) | 96 (77.42) | **0.05**^d^ | 2.500 (1.500, 33.000) | **<0.001** |
|  |  |  | M | 1 (4.00) | 24 (96.00) |  | 33.000 (33.000, 33.000) |  |
|  |  | D91N/Y/G^a^ | W | 29 (24.37) | 90 (75.63) | **0.003**^d^ | 2.000 (1.500, 33.000) | **<0.001** |
|  |  |  | M | 0 (0.00) | 30 (100.00) |  | 33.000 (33.000, 33.000) |  |
| MZ | *rdxA* | R16C/H/Q^a^ | W | 27 (22.50) | 93 (77.50) | 0.16^d^ | 257.000 (12.000, 257.000) | 0.88 |
|  |  |  | M | 3 (12.50) | 21 (87.50) |  | 192.500 (32.000, 257.000) |  |
|  |  | L209A/C/D/Y^a^ | W | 30 (21.90) | 107 (78.10) | 0.35^d^ | 257.000 (16.000, 257.000) | 0.23 |
|  |  |  | M | 0 (0.00) | 7 (100.00) |  | 257.000 (96.000, 257.000) |  |
|  |  | A118D/R/S/T^a^ | W | 27 (19.85) | 109 (80.15) | 1.00 ^d^ | 257.000 (32.000, 257.000) | 0.36 |
|  |  |  | M | 3 (21.43) | 11 (78.57) |  | 128.000 (9.000, 257.000) |  |
|  |  | K64N/R^a^ | W | 29 (20.71) | 111 (79.29) | 1.00^d^ | 257.000 (24.000, 257.000) | 0.27 |
|  |  |  | M | 1 (14.29) | 6 (85.71) |  | 257.000 (98.000, 257.000) |  |
|  |  | A68G/H/M/R/S/T/V^a^ | W | 27 (22.31) | 94 (77.69) | 0.19^c^ | 257.000 (12.000, 257.000) | **0.04** |
|  |  |  | M | 3 (11.11) | 24 (88.89) |  | 257.000 (128.000, 257.000) |  |
|  |  | A67C/Q/V^a^ | W | 30 (21.90) | 107 (78.10) | 0.20^d^ | 257.000 (15.000, 257.000) | 0.43 |
|  |  |  | M | 0 (0.00) | 9 (100.00) |  | 257.000 (96.000, 257.000) |  |
|  |  | M21A/I/L^a^ | W | 24 (20.17) | 95 (79.83) | 0.72^c^ | 257.000 (20.000, 257.000) | 0.59 |
|  |  |  | M | 5 (17.24) | 24 (82.76) |  | 257.000 (32.000, 257.000) |  |
|  | *frxA* | R206C/H/P^a^ | W | 29 (20.71) | 111 (79.29) | 0.35^d^ | 257.000 (32.000, 257.000) | 0.6 |
|  |  |  | M | 0 (0.00) | 7 (100.00) |  | 257.000 (64.000, 257.000) |  |
|  |  | R106G/K/L^a^ | W | 30 (21.28) | 111 (78.72) | 0.21^d^ | 257.000 (32.000, 257.000) | **0.03** |
|  |  |  | M | 0 (0.00) | 10 (100.00) |  | 257.000 (257.000, 257.000) |  |
|  |  | M66K/L^a^ | W | 28 (20.00) | 112 (80.00) | 1.00^d^ | 257.000 (40.000, 257.000) | 0.86 |
|  |  |  | M | 2 (20.00) | 8 (80.00) |  | 192.500 (25.000, 257.000) |  |
|  |  | A32G/T/V^a^ | W | 15 (17.05) | 73 (82.95) | 0.45^c^ | 257.000 (48.000, 257.000) | 0.25 |
|  |  |  | M | 12 (22.22) | 42 (77.87) |  | 256.000 (32.000, 257.000) |  |
|  |  | S43A/C/H/T/V^a^ | W | 0 (0.00) | 8 (100.00) | 0.21^d^ | 257.000 (208.250, 257.000) | 0.09 |
|  |  |  | M | 31 (21.68) | 112 (78.32) |  | 257.000 (32.000, 257.000) |  |
|  |  | D62A/L/S/V/Y^a^ | W | 28 (20.44) | 109 (79.56) | 1.00^c^ | 257.000 (32.000, 257.000) | 0.72 |
|  |  |  | M | 2 (16.67) | 10 (83.33) |  | 257.000 (36.000, 257.000) |  |
|  |  | A70G/I/K/L/S/V^a^ | W | 26 (20.80) | 99 (79.20) | 0.53^c^ | 257.000 (32.000, 257.000) | 0.75 |
|  |  |  | M | 2 (11.11) | 16 (88.89) |  | 257.000 (48.000, 257.000) |  |
| TC | *16S rRNA* | A926C/G/T^b^ | W | 115 (87.79) | 16 (12.21) | **0.006**^c^ | 0.500 (0.250, 0.750) | **0.001** |
|  |  |  | M | 13 (61.90) | 8 (38.10) |  | 1.000 (0.500, 4.500) |  |
|  |  | A928C^b^ | W | 123 (86.62) | 19 (13.38) | **0.01**^c^ | 0.500 (0.250, 0.875) | **<0.001** |
|  |  |  | M | 5 (50.00) | 5 (50.00) |  | 1.500 (0.938, 4.250) |  |

1. Mutations are defined using *H.pylori 26695* as the reference genome and reported following standard recommendations in molecular diagnostics from the Human Genome Variation Society. For genes coding proteins, mutations in codons were described. A substitution with missense change (e.g. K229R) is described by the residue in the wild type strain, the sequence position and the residue in the mutant type strain. In-frame insertions (e.g. K464_D465insE/K/D) are described using ‘ins’ after an indication of the residue flanking the insertion site, separated by ‘_’ (underscore) and followed by a description of the residue inserted. Single-letter abbreviations for amino acid residues in protein sequences include A for alanine, C for cysteine, D for aspartic acid, E for glutamic acid, F for phenylalanine, G for glycine, H for histidine, I for isoleucine, K for lysine, L for leucine, M for methionine, N for asparagine, P for proline, Q for glutamine, R for arginine, S for serine, T for threonine, V for valine, W for tryptophan, and Y for tyrosine.
2. For genes coding RNA, the base positions of the mutations were described. Nucleotide residues in DNA sequences are represented by heterocyclic bases as A for adenine, C for cytosine, G for guanine, and T for thymine.
3. Chi-square test for the comparison of resistance rate between wild and mutant type strains.
4. Fisher’s exact test for the comparison of resistance rate between wild and mutant type strains.
5. Man-Whitney test for the comparison of MIC between wild and mutant type strains.

AC, amoxicillin, CH, clarithromycin, LE, levofloxacin, MZ, metronidazole, TC, tetracycline, W, wild type, M, Mutant type, MIC, minimum inhibitory concentration.
